# Pteridine-2,4-diamine derivatives as radical scavengers and inhibitors of lipoxygenase that can possess anti-inflammatory properties

**DOI:** 10.4155/fmc.15.104

**Published:** 2015-10-01

**Authors:** Eleni Pontiki, Dimitra Hadjipavlou-Litina, Alexandros Patsilinakos, Trang M Tran, Charles M Marson

**Affiliations:** 1 Christopher Ingold Laboratories, Department of Chemistry, University College London, 20 Gordon Street, London WC1H OAJ, UK; 2 Department of Pharmaceutical Chemistry, Faculty of Health Sciences, School of Pharmacy, Aristotle University of Thessaloniki, Thessaloniki, 54124, Greece; 3 Rome Center for Molecular Design, Dipartimento di Chimica e Tecnologie del Farmaco, Sapienza University of Rome, 00185 Rome, Italy

## Abstract

**Background:**

Reactive oxygen species are associated with inflammation implicated in cancer, atherosclerosis and autoimmune diseases. The complex nature of inflammation and of oxidative stress suggests that dual-target agents may be effective in combating diseases involving reactive oxygen species.

**Results:**

A novel series of *N*-substituted 2,4-diaminopteridines has been synthesized and evaluated as antioxidants in several assays. Many exhibited potent lipid antioxidant properties, and some are inhibitors of soybean lipoxygenase, IC_50_ values extending down to 100 nM for both targets. Several pteridine derivatives showed efficacy at 0.01 mmol/kg with little tissue damage in a rat model of colitis. 2-(4-methylpiperazin-1-yl)-*N*-(thiophen-2-ylmethyl)pteridin-4-amine (**18f**) at 0.01 mmol/kg exhibited potent anti-inflammatory activity (reduction by 41%).

**Conclusion:**

The 2,4-diaminopteridine core represents a new scaffold for lipoxygenase inhibition as well as sustaining anti-inflammatory properties.

Oxidative stress is closely associated with chronic inflammation and plays a crucial role in cancer [1], dyslipidemia [2], atherosclerosis [3] and autoimmune diseases such as systemic lupus erythematosus and rheumatoid arthritis [4]. In many diseases, the rate of production of reactive oxygen species (ROS) is increased compared with normal levels of ROS [5,6]. ROS are produced during the inflammatory process by phagocytic leukocytes that invade the tissue. Under conditions of oxidative stress, ROS including superoxide anion, hydroxyl radical and hydrogen peroxide and their reactive products can attack various biological macromolecules (e.g., proteins, enzymes, DNA and lipids) resulting in DNA mutations, lipid peroxidation and protein oxidation, [7] or they may indirectly interfere with mechanisms of DNA repair [8]. Thus, ROS activity consists of a mixture of deleterious and beneficial roles, depending on the type, concentration and location of the species involved. The breadth of those factors suggests that focused targeting of ROS, probably in a dual-target or multiple-target approach, could be of therapeutic value.

Reactive oxygen species are centrally involved in the cyclooxygenase (COX)- and lipoxygenase (LOX)-mediated conversion of arachidonic acid (AA) into proinflammatory intermediates [9,10]. LOX exerts its biological role via a carbon-centered radical mechanism. LOX metabolism results in many bioregulatory molecules such as leukotrienes, lipoxins and hepoxylins, mediators in the pathophysiology of variety of diseases such as rheumatoid arthritis, bronchial asthma, psoriasis, cancer and other inflammatory diseases [11].

In the search for new antioxidants and anti-inflammatory agents, the pteridine ring (Figure 1) was selected for study for several reasons: many naturally-occurring derivatives possess important biological activity, including the coenzymes 5,6,7,8-tetrahydrobiopterin and the pterins; the ring system has low toxicity; synthetic pteridine derivatives show a wide range of clinically useful properties, for example, as antioxidants, immunosuppressants, and anti-inflammatory and anticancer agents. Consequently, multitarget properties were deemed likely, as were the discovery of new agents for a range of diseases, and also the use of pteridine derivatives to probe the putative targets of diseases.

Substituted pteridines are known to act upon a wide range of targets of therapeutic potential. Thus, a 6,7-disubstituted-2,4-diaminopteridine is a PI3 kinase inhibitor with potential for treatment of myocardial infarction involving ischemia reperfusion injury [12]. 2-Amino-4-piperazin-1-yl-6-(3,4-dimethoxyphenyl)-pteridines have been shown to possess immunosuppressive and anti-inflammatory properties [13], and analogues of the potent phosphodiesterase-4 inhibitor 7-benzylamino-6-chloro-2-piperazin-1-yl-4-pyrrolidin-1-ylpteridine which inhibits growth in tumor cell lines required a 2-piperazin-1-yl substituent for optimal potency [14]. Some 2-amino-4-(*N,N*-diarylmethyl)-6-arylpteridines inhibit neuronal nitric oxide synthase [15] and some 2,4-diamino-4,6-diarylpteridines inhibit inducible or inflammatory nitric oxide synthase [16]. Several 2,4-diamino-6-arylaminomethylpteridines are potent inhibitors of parasitic pteridine reductases, and have promising antiparasitic potential [17,18]. Adequate dietary content of folic acid (pteroyl l-glutamic acid; Figure 1) is a requirement for human health and on which DNA synthesis, DNA repair and DNA methylation depend [19].

In addition to the treatment of a variety of cancers, the antifolate methotrexate reduces inflammation in the bowel and is used for treating Crohn’s disease, ulcerative colitis, rheumatoid arthritis, psoriasis and other autoimmune diseases. Therefore, novel compounds bearing the pteridine ring could exhibit anti-inflammatory and antioxidant activities and have potential as therapy for a wide range of diseases involving inflammation. The 2,4-diaminopteridine core of methotrexate (Figure 1), was used as the scaffold in this study. Alkylamino and dialkylamino substituted 2,4-diaminopteridines were investigated since many have a range of drug-like clogP values (1.0–4.0, Table 1) and because synthesis should afford the required structural diversity. We describe here the synthesis and preliminary evaluation of some novel *N*-alkylated 2,4-diaminopteridine derivatives as dual-target agents through their radical scavenging properties, inhibition of lipoxygenase and *in vivo* anti-inflammatory activity.

## Results & discussion

### Chemistry

**Figure 2:**
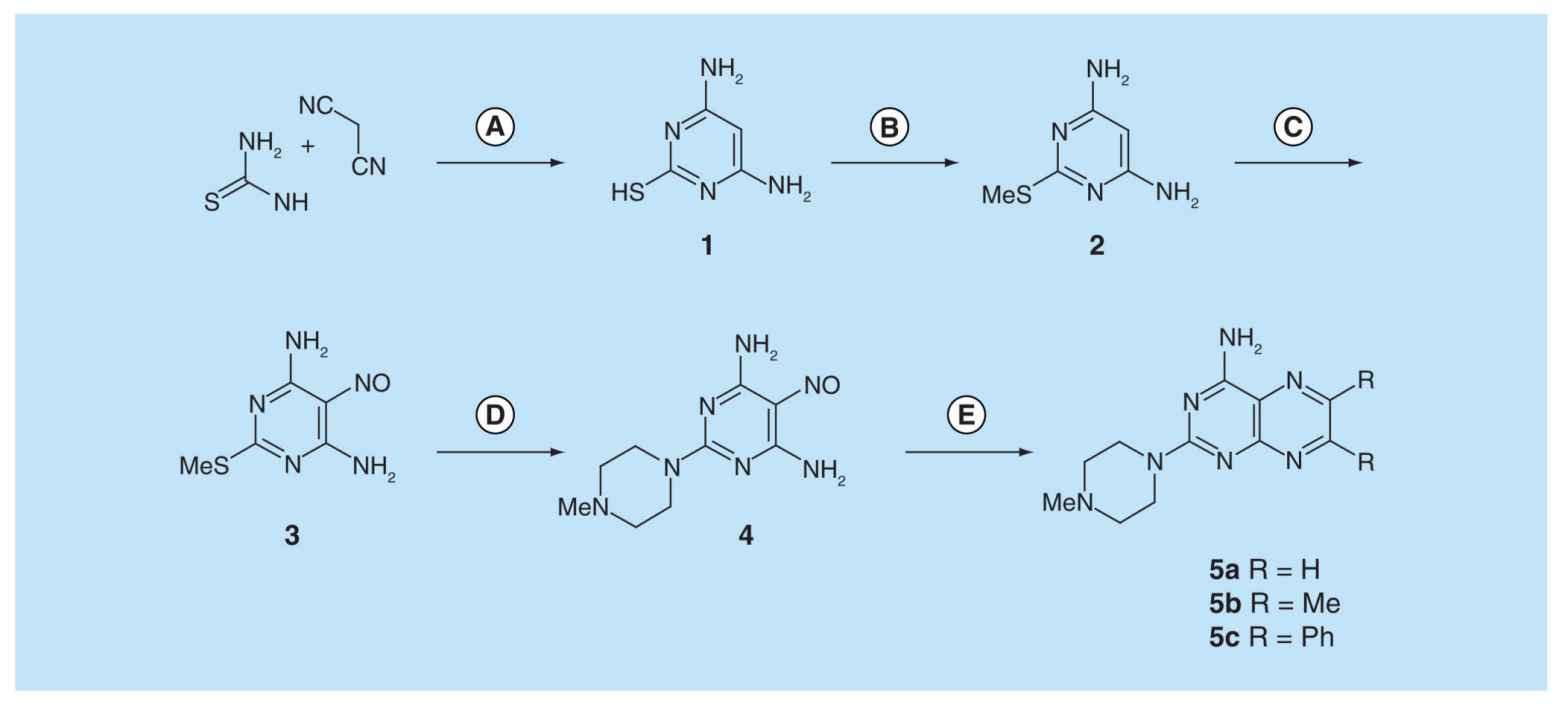
Synthesis of pteridines (5). Reagents and conditions: **(A)** Na, ethanol, reflux. 2 h; **(B)** methyl iodide, ethanol, reflux 1.5 h; **(C)** NaNO_2_ aqueous acetic acid, 0°C, 2 h then 4°C, 16h; **(D)** 1-methylpiperazine ethanol, reflux 0.75 h then add water and reflux 0.75 h; **(E)** sodium dithionite then aqueous 40% glyoxal (**5a**) or butane-2,3-dione (**5b**, or benzil [**5c**]), reflux 8 h, 18 h and 24 h, respectively.

Routes to 2,4-diaminopteridine derivatives with no substituents at the 6- or 7-postions are sparse. In particular, very few such compounds contain a tertiary amine at the 2-position (Figure 2). Two relevant 6,7-disubstituted pteridines had been prepared by reaction of 4-amino-2-methylthio-6,7-diphenylpteridine with morpholine and with piperidine to give the corresponding 2-substituted 4-amino-6,7-diphenyl-pteridines, but a large excess of the amine was used as the solvent, and it was not known whether this route would permit the introduction of amines other than amino at the 4-position, since only 4,5-diamino-2-methylthiopyrimidine has been used as the early precursor in this route [20]. Accordingly, it was decided to introduce the desired 2,4-diamino substitution prior to the formation of the pteridine ring. Since dimethylamine, pyrrolidine and piperidine had been shown to displace the 2-methylthio group of 4,6-diamino-2-methylthio-5-nitrosopyrimidine (3) [21], the route envisaged here involved 5-nitrosation, 2-amination, then reduction and cyclization to give the pteridine ring system (Figure 2).

4,6-diamino-2-thiopyrimidine (**1**), prepared by condensing thiourea and malononitrile in the presence of sodium ethoxide [22], was *S*-methylated to give 4,6-diamino-2-methylthiopyrimidine (**2**) by heating with ethanolic methyl iodide at reflux [20]. In a modification of the literature procedure [23], treatment of **2** in aqueous sodium nitrite containing acetic acid afforded the nitrosopyrimidine **3** (95%) which with 1-methylpiperazine in ethanol at reflux was converted into the 2-(4-methylpiperazin-1-yl)-5-nitrosopyrimidine 4 (57%). Reduction of **4** with aqueous sodium dithionite afforded the corresponding tetraamine which without isolation was treated with aqueous 40% glyoxal and then heated at reflux to give 2-(4-methylpiperazin-1-yl) pteridin-4-amine (**5a**). In the same way, butane-2,3-dione and benzil afforded the corresponding pteridines **5b** (68%) and **5c** (59%).

**Figure 3:**
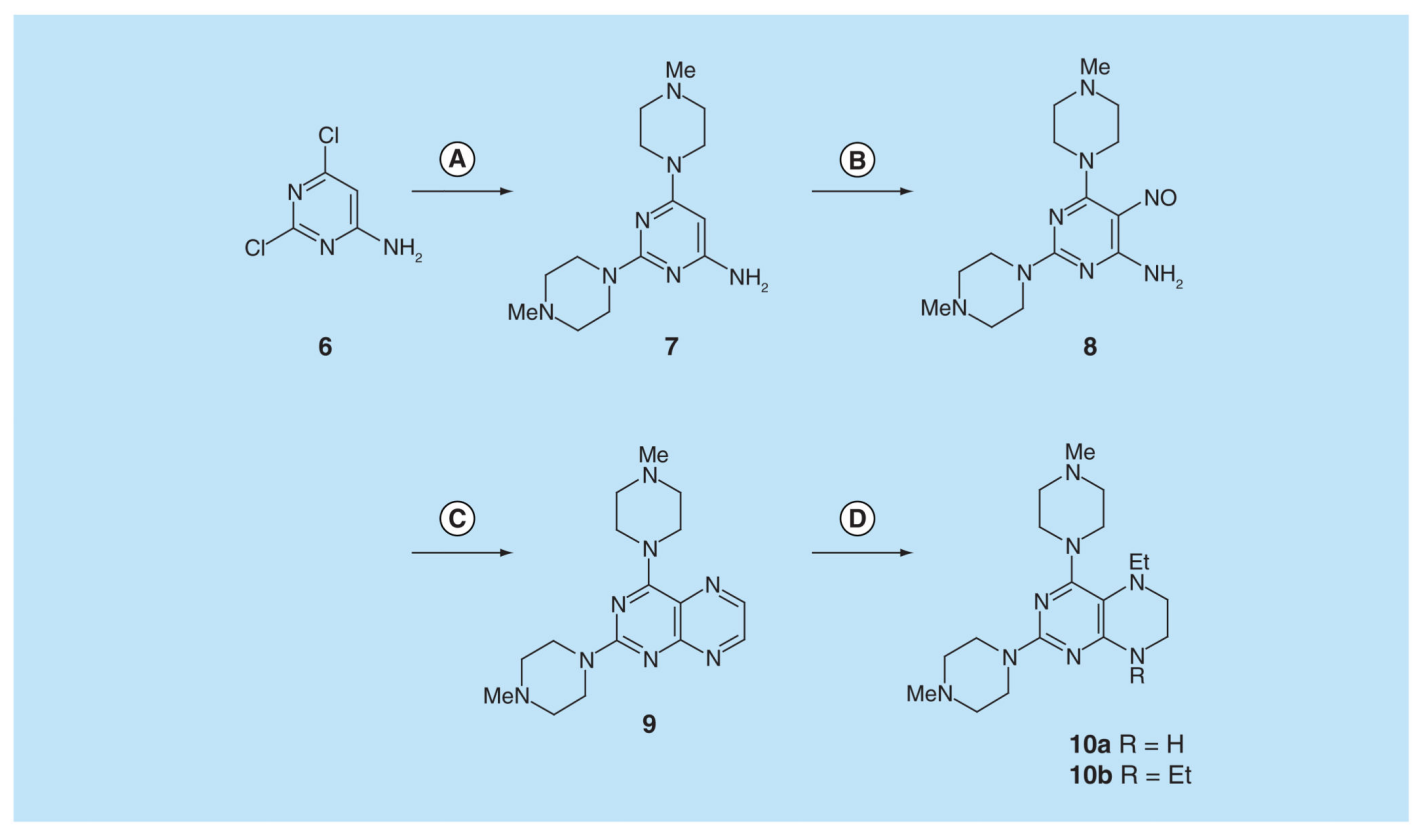
Synthesis of 5,6,7,8-tetrahydropteridines (10). Reagents and conditions: **(A)** 1-methylpiperazine, reflux, 18 h; **(B)** NaNO_2_ aqueous acetic acid, 0°C, 3 h; **(C)** sodium dithionite then aqueous 40% glyoxal, reflux 7 h; **(D)** NaHB(OAc)_3_ (9 eq for **10a** and 1 eq for **10b**), acetic acid 48 h, 20°C.

**Figure 4:**
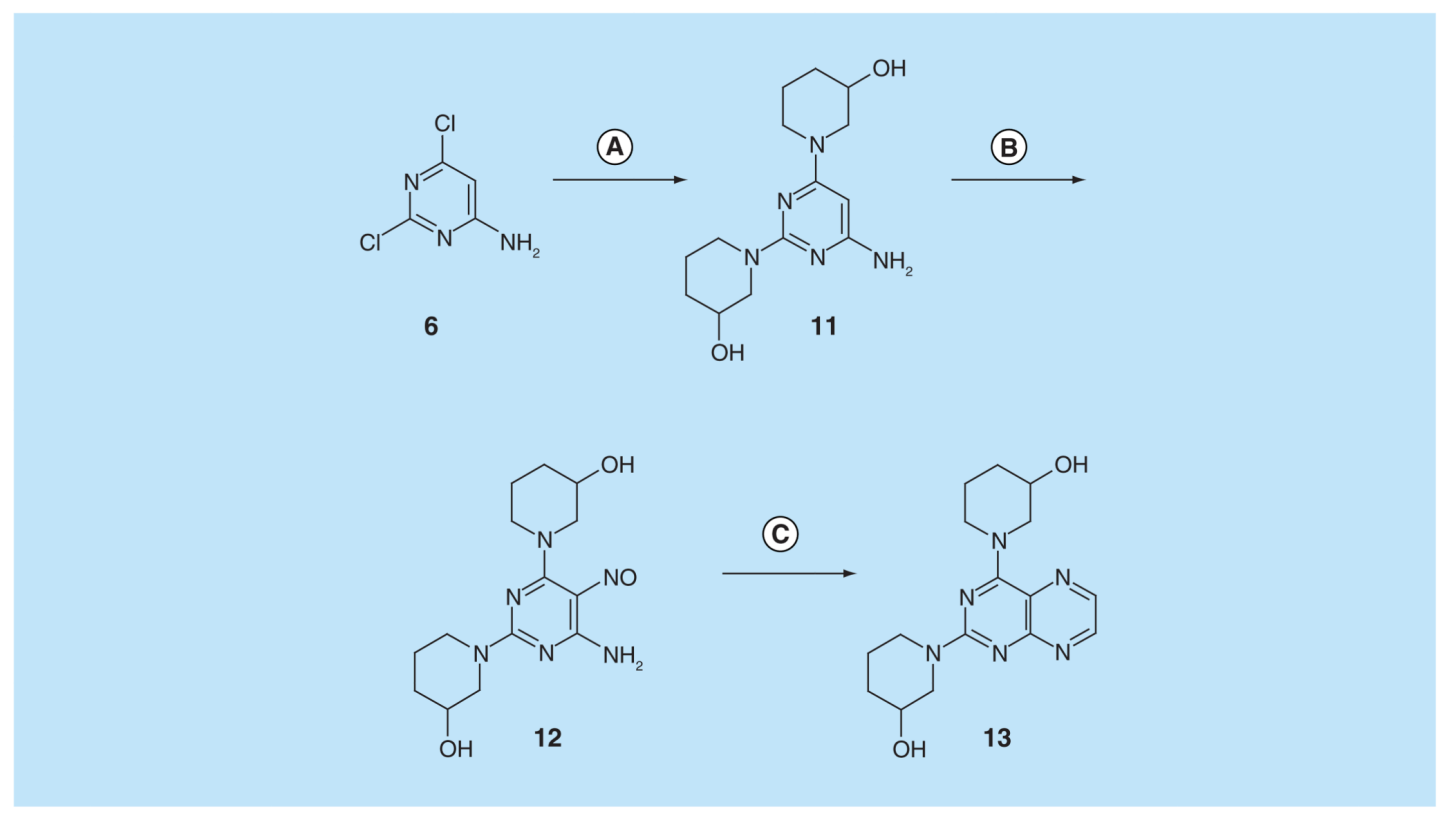
Synthesis of pteridine 13. Reagents and conditions: **(A)** 3-hydroxypiperazine, reflux 5 h; **(B)** NaNO_2_ aqueous acetic acid 0°C, 3 h; **(C)** sodium dithionite then aqueous 40% glyoxal, reflux 6 h.

For 2,4-diaminopteridine derivatives bearing identical 2- and 4-substitution, 6-amino-2,4-dichloropyrimidine was heated at reflux with the appropriate secondary amine to give the triamino-substituted pyrimidines **7** (71%) and **11** (89%) (Figures 3 & 4). Using the previous procedure, nitrosation afforded **8** (98%) and **12** (71%) which with aqueous sodium dithionite afforded the tetraamines, and again without isolation those were treated with aqueous 40% glyoxal to afford the corresponding pteridine derivatives **9** (48%) and **13** (41%). The diol **13** was obtained as a mixture of two diastereoisomers that were not separated. Reduction using sodium triacetoxyborohydride (9 mole equivalents) in glacial acetic acid gave the 5-ethyl-5,6,7,8-tetrahydropteridine **10a** (29%), after column chromatography. However, when one mole equivalent of triacetoxyborohydride was used, the 5,8-diethyl-5,6,7,8-tetrahydropteridine **10b** (46%) was obtained, after column chromatography.

**Figure 5:**
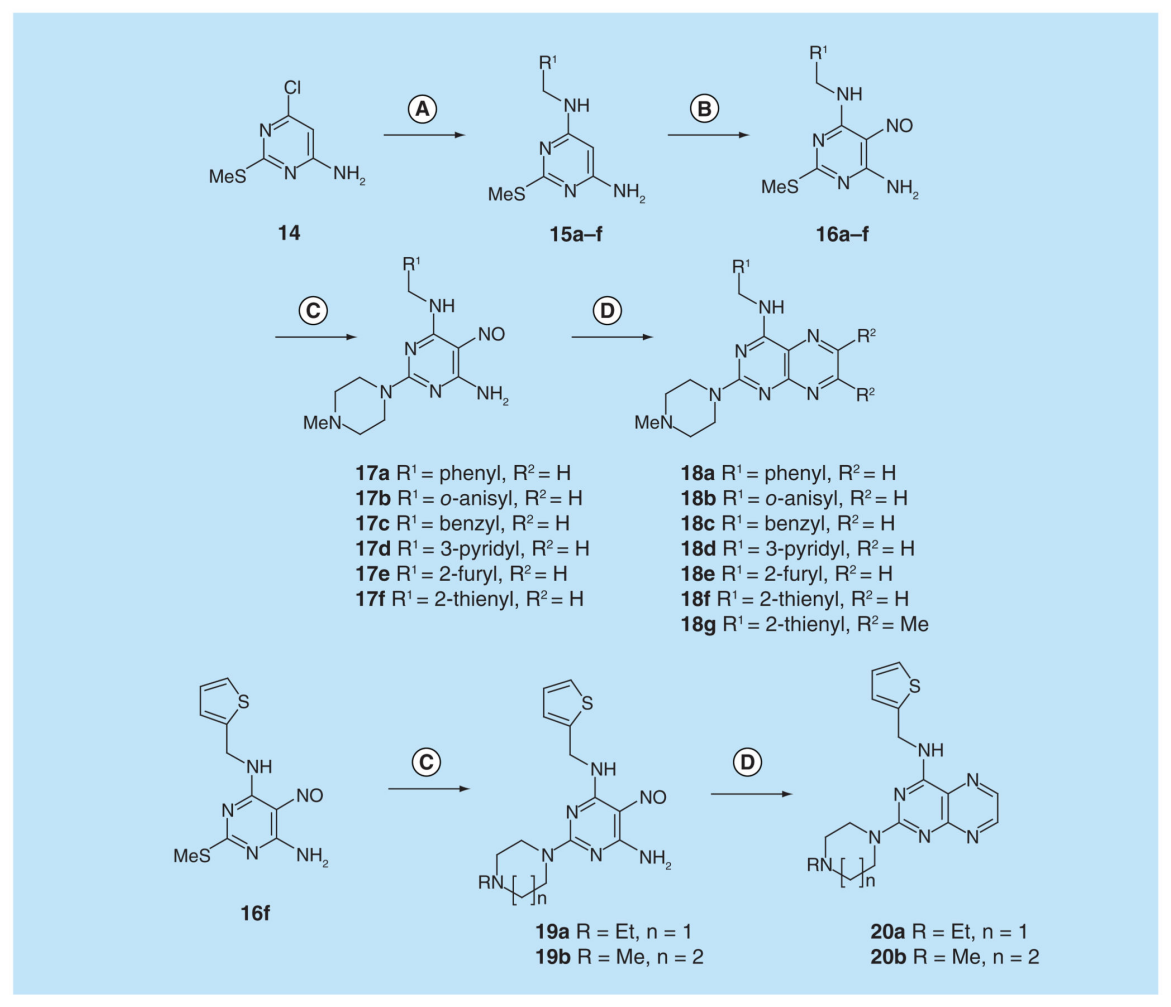
Synthesis of pteridines 18 and 20. Reagents and conditions: **(A)** R^1^CH_2_NH_2_ diglyme, reflux, 5 h; **(B)** NaNO_2_ aqueous acetic acid, 0°C, 2 h then 4°C, 16 h; **(C)** 1-methylpiperazine or 4-methyl-1,4-diazepane, ethanol, reflux, 2h then add water and reflux 1 h; **(D)** sodium dithionite then aqueous 40% glyoxal or butane-2.3-dione, reflux.

For dissimilar substitution at the 2- and 4-positions of pteridine-2,4-diamine derivatives, a stepwise introduction of those substituents was required. 2,4-dichloropteridine [24] and its derivatives [25] react regioselectively at the 4-position with one equivalent of amine, which makes this approach unsuitable for preparing a range of pteridines with the same 2-amino substituent. Additionally, the use of 2,4,6,7-tetrachloropteridine for related successive displacements with different amines, while succinct, afforded extensive mixtures and with isolation of desired single regioisomers usually in low yields, after chromatography [14]. Since the formation of pteridines from 5-nitrosopyrimidines had proved robust and had delivered single regioisomers, this approach was adopted, in which the 4-amino group would be introduced by displacement of 4-amino-6-chloro-2-(methylthio)pyrimidine **14** (Figure 5). However, displacement of the chloro group had been reported only for pyrimidine **14**, using dimethylamine to install a 4-dimethylamino group [26]. 4,6-Diamino-2-methylthiopyrimdines such as **15** would be required, and would be obtained by displacement of **14** with benzylamine derivatives or with heteroarylmethylamines (Figure 5). Displacement of the 2-methylthio group in **16** by a secondary amine would give the unsymmetrically substituted 2,4,6-triamino-5-nitrosopyrimidines, and hence the corresponding pteridines **18**.

4-Amino-6-chloro-2-(methylthio)pyrimidine **14** reacted with a variety of amines (2.1 equivalent) in diglyme at reflux to give the corresponding pyrimidines **15a–f** [27] which underwent 5-nitrosation with acidic aqueous sodium nitrite (Figure 5) [23]. The 2-methylthio group of the resulting 5-nitrosopyrimdines **16a–f** underwent displacement with 1-methylpiperazine or 4-methyl-1,4-diazepane in ethanol at reflux to give the corresponding 2-amino derivatives **17a–f**. Those were subjected to the above reduction with sodium dithionite followed by condensation with aqueous 40% glyoxal to give the pteridine derivatives **18a–g**. Compounds **20a** and **20b** were prepared analogously, the latter by using 4-methyl-1,4-diazepane in ethanol at reflux to give **19b**. ^1^H NMR spectra of the nitroso compounds **16, 17** and **19** in chloroform are consistent with the presence of two rotamers arising from hydrogen bonding between the C-5 nitroso oxygen atom and the adjacent NH hydrogen atoms at C-4, and also with an NH hydrogen atom at C-6, as established for related pyrimidines [28–30].

**Figure 6:**
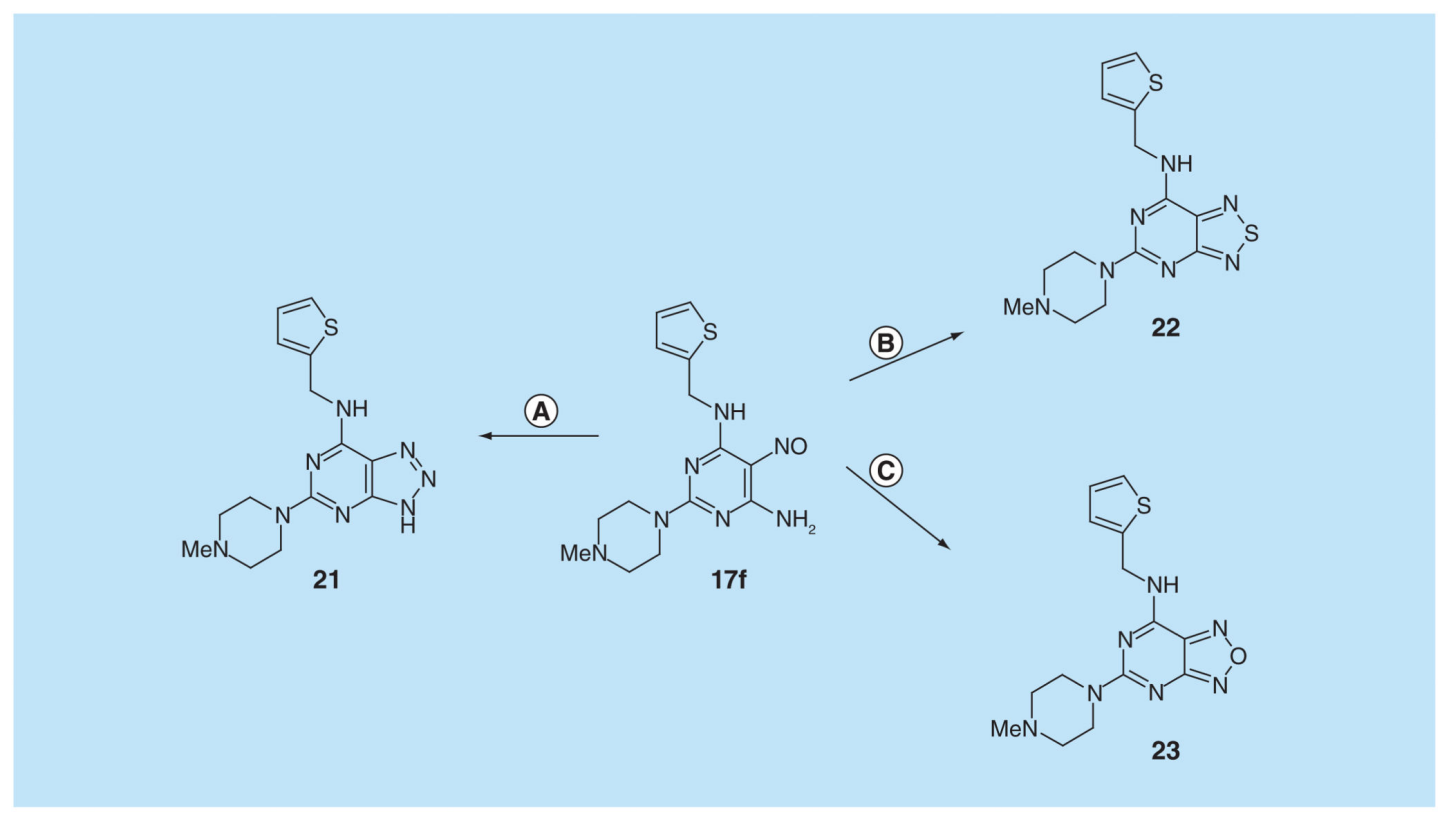
Synthesis of pteridine analogs 21, 22 and 23. Reagents and conditions: **(A)** H_2_, 10% Pd on C, ethanol; then NaNO_2_, glacial acetic acid, 90°C, 2 h; **(B)** sodium thiosulfate pentahydrate, aq. 20% acetic acid 90°C, 1.5 h; **(C)** lead tetraacetate, acetic acid, 20°C, 4 h.

A representative 5-nitrosopyrimidine, **17f**, was transformed into three 6,5-fused systems (Figure 6) in a brief survey of the relevance of the fused pyrazine ring in the pteridine series. The triazolo[4,5-*d*]pyrimidine **21** was obtained by hydrogenation of **17f** to the corresponding amine, nitrosation and subsequent ring closure at 90°C, following standard methods [31]. The 5-nitrosopyrimdine **17f** was also converted into **22** by sodium thiosulfate [32], and into **23** by lead tetraacetate [33]. Synthetic procedures for the new pteridine derivatives are described in detail in the Supplementary Information. All new compounds showed spectroscopic data consistent with the structures proposed. The purity of tested compounds was assessed as at least 95% by HPLC–MS, unless otherwise indicated.

## *In vitro* lipoxygenase inhibition

The substituted pteridines prepared were assayed for inhibition of soybean lipoxygenase (Table 1) [34,35] (Supplementary Information). Many studies have used readily obtainable soybean lipoxygenase, which is a homologue of mammalian lipoxygenase and well examined [36,37]. The availability of soybean LOX and its well-characterized crystal structure [38] led to its use in this study.

In the soybean LOX inhibition assay (Table 1) the three 4-amino-2-(4-methylpiperazin-1-yl)pteridines (entries 1–3) showed only weak inhibition, with increasing bulk of 6,7-substituents not enhancing potency; similarly, the 6,7-dimethyl substitution in compound **18g** lowered LOX inhibitory activity, the 6,7-unsubstituted pteridine **18f** exhibiting the greater potency. Substitution at the 4-amino group generally increased potency, and within the (hetero)arylmethyl series, polarity in a substituent (**18b**) or in the appended ring (**18d**) further increased potency, in the latter case vary greatly. However, the 4-(hetero)arylmethyl series and the 4-(phenethylamine) pteridine **18c** showed only moderate potency. Comparison of **18f** with **20a** and **20b** shows that both 2-(4-ethylpiperazin-1-yl) and 1-ethyl-1,4-diazepanyl groups conferred lower potency compared with a 2-(4-methyl-piperazin-1-yl) moiety. Of the compounds containing a 4-(4-methylpiperazin-1-yl) substituent (entries 4–6), **9** was the most potent pteridine, and **10a** the most potent 5,6,7,8-tetrahydropteridine, both having IC_50_ = 5 μM for inhibition of LOX. However, 5,8-diethyl substitution, as in **10b**, was less well tolerated than the 8-unsubstituted **10a**. A limitation on ring tolerance was also identified; the 3-hydroxypiperidin-1-yl 2,4-disubstitution in **13** conferred some tenfold less potency than the preferred 2,4-di-(4-methylpiperazin-1-yl) substitution present in **9**. The pyrimido[4,5-*d*]azoles **21–23**, containing the standard 2- and 4-substituents, were only moderately potent LOX inhibitors. Lastly, the lipophilicities of the pteridines were considered since a correlation of lipophilicity with LOX inhibition has been reported [39] in other studies but was not detected here [34]. Lipophilicity, as assessed using calculated clogP values of the substituted pteridines (Table 1), does not appear to inhibit significantly *in vitro* LOX, for example, pteridine **9** and the 5,6,7,8-tetrahydropteridine **10a** showing equipotent inhibition of LOX (IC_50_ = 5 μM) although their clogP values differ by 2.5; however, the most potent LOX inhibitor identified, **18d** (IC_50_ = 0.10 μM), does have a relatively low clogP (0.92).

The LOX inhibition data (Table 1) show that the 4-amino substituent plays a crucial role in determining the potency of the substituted pteridine. Thus, although a 4-benzylamino group (entry 8) is almost equipotent to an unsubstituted amino group (entry 5a), a 4-(4-methylpiperazin-1-yl) group (entry 4) shows some tenfold increase in potency. However, a 3-hydroxypiperidin-1-yl moiety (entry 7) affords only moderate LOX inhibition. A nitrogen atom in the 4-substituent can confer excellent potency (entry 11). Soybean LOX is able to accommodate the very rigid 4-methylpiperazin-1-yl group present in **9** (IC_50_ = 5.0 μM) although the flexible (3-pyridylmethyl)amino group present in **18d** (IC_50_ = 0.10 μM) confers much greater potency. That both substituents are proton acceptors is consistent, in each case, with the distal nitrogen atom engaging in hydrogen bonding. Entries 5 and 8 (Table 1) suggest that in regard to LOX inhibition some steric bulk is tolerated at the 5-position, but is much less well tolerated at the 8-position of the pteridine ring. Although no definite conclusions can currently be drawn, tolerance of some substituents at the 6- and/or 7-positions seems likely.

## Molecular modeling of LOX

**Figure 7:**
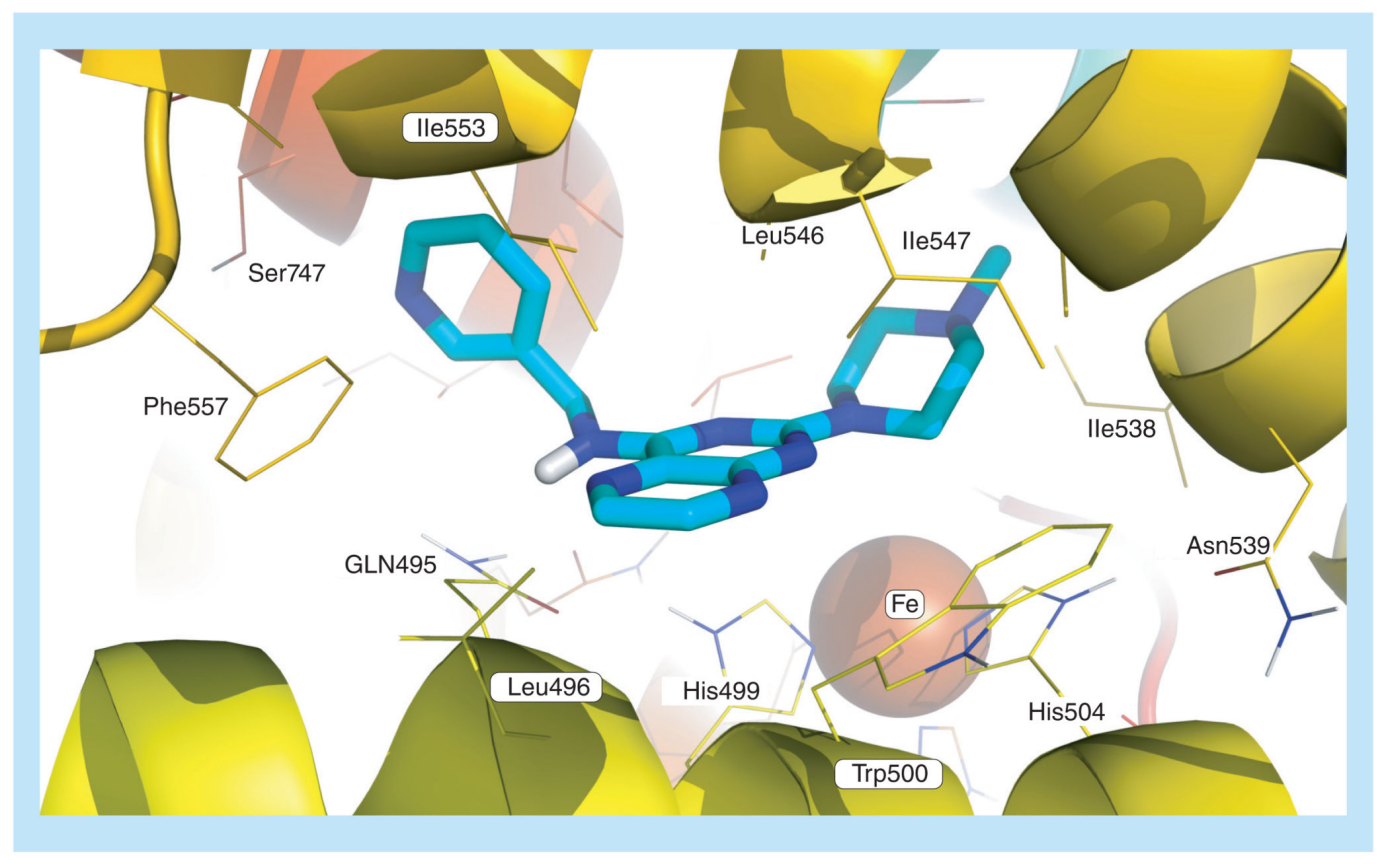
Docking pose of pteridine 18d (depicted in turquoise) bound to soybean lipoxygenase (LOX-1) derived by modification of PDB code: 3PZW. Energy minimizations were carried out using the AMBER99SB-ILDN force field [41] with GROMACS as the molecular simulation toolkit [42]. AutoDock Vina (1.1.2) [41] was used for docking. Iron is rendered as a brown sphere. Prepared using PyMOL, this figure represents the preferred pose according to scoring function.

Being the most potent inhibitor of soybean LOX of the compounds studied and also possessing efficacy as an antioxidant, pteridine derivative **18d** was selected for *in silico* docking. The molecular modeling study performed (see Supplementary Information for details) provided useful interpretation of the experimental results. The preferred docking orientation for compound **18d** is shown in Figure 7. The binding of **18d** to soybean LOX (PDB code: 3PZW) has a higher AutoDock Vina score (-8.5 kcal/mol) than any of the other pteridines docked. Pteridine **18d** is able to accommodate the extensively hydrophobic cavity close to the active site, incorporating Ile552, Ile553, Ile538 and Leu546 among other residues. Ile553 and especially Leu496 are proximate to the hydrophobic 6,7-flank of the pteridine ring, Ile553 also extending to the hydrophobic C4-C6 region of the pyridine ring in **18d**. The increased potency of **18d** over its phenyl analog **18a** is considered to be due to hydrogen binding, perhaps to Ser747. The simplest explanation is that the extension scaffold of **18d**into the hydrophobic domain blocks approach of substrates to the active site, and hence prevents oxidation by soybean LOX. The docking simulations of NDGA and **18d** show a common pattern of interaction with LOX (Supplementary Figure 1), the terminal rings and central core of each compound showing appreciable overlap. Additionally, Ser747 is engaged in hydrogen bonding with the 3-hydroxyl group of the catechol unit of NDGA, and also with the pyridine nitrogen atom of **18d**. The relatively weak antioxidant properties of **18d** (Table 2) are also consistent with the main mode of action of LOX inhibition being other than by diminishing general ROS concentrations and hence antioxidant activity [40]. Preliminary screening tests of the pteridines against COX did not present any significant inhibition.

## *In vitro* antioxidant activity

Previous work has shown that pterins, their dihydro- and their tetrahydro-derivatives can each be antioxidants or pro-oxidants, depending on the particular conditions [43]. In the present study, several assays were used to assess *in vitro* antioxidant activity in order to obtain representative information; each method involves the generation of a different radical. The three assays chosen measured *in vitro* antioxidant activity in terms of: reduction of the stable free radical 1,1-diphenyl-2-picrylhydrazyl (DPPH), whose oxidized form possesses an absorption maximum at 517 nm; hydroxyl radical scavenging activity; extent of reduction of the water-soluble 2,2′-azo-bis(2-amidinopropane) dihydrochloride (AAPH) and inhibition of soybean lipoxygenase (Supplementary Information).

The pteridine derivatives were evaluated for their antioxidant activity (Table 2) and compared with that of nordihydroguaiaretic acid (NDGA), the reference compound [39]. Although most compounds at 100 μM did not show significant reducing ability, key exceptions were the potent 5-ethyl-5,6,7,8-tetrahydropteridine **10a** (81%) and very potent 5,8-diethyl-5,6,7,8-tetrahydropteridine **10b** (97%) (Table 2), the latter being more effective than the reference compound.

Competition of the novel pteridine derivatives with dimethyl sulfoxide for hydroxyl radicals was measured. Hydroxyl radicals were generated using the Fe3+/ascorbic acid system and expressed as a percentage inhibition of formaldehyde production in the presence of each pteridine derivative at 100 μM (Table 2) [34]. Pteridine derivatives **5a, 10b** and **18g** strongly inhibited the oxidation of dimethyl sulfoxide (33 mM). The majority of the derivatives were excellent scavengers of hydroxyl radicals with activity higher than the reference compound 6-hydroxy-2,5,7,8-tetramethylchroman-2-carboxylic acid (Trolox).

Azo compounds that generate free radicals through spontaneous thermal decomposition are useful for *in vitro* studies of free radical production. The water-soluble AAPH has been extensively used as a clean and controllable source of thermally produced alkylperoxyl free radicals [44]. In this assay, compound **18g** (IC_50_ = 0.1 μM) was the most potent in protecting against lipid peroxidation; next, and almost equipotent, were the pteridines **13** and **18e**, and the triazolo[4,5-*d*]pyrimidine **22** and the oxadiazole[3,4-*d*]pyrimidin-4-amine **24** (each of approximate IC_50_ = 0.3 μM). The 4-(thiophen-2-yl)methylamino-substituted pteridine derivatives showed a range of three orders of magnitude, compound **18g** showing by far the greatest protection against lipid peroxidation, the 2-(4-ethylpiperazin-1-yl) derivative **20a** being moderate, the 1,4-diazepane derivative **20b** being poor and the 2-(4-methylpiper-azin-1-yl) derivative **18f** being very weak. Evidently, the presence of 6,7-dimethyl groups on the pteridine ring greatly enhances protection against lipid peroxidation; the nature of the alkyl group and ring size on the 2-substituent has some, but much less, effect. Of the 4-aminopteridines, the 6,7-diphenyl derivative **5c** was by far the most potent inhibitor, whereas of the 2,4-bis(4-methylpiperazin-1-yl) derivatives, **13** is more than twice as potent as the 2,4-(3-hydroxydipiperidin-1-yl derivative **9**. Reduction of the pteridine ring in compound **9** to the corresponding 5,6,7,8-tetrahy-dropteridines **10a** and **10b** decreased protection of lipid peroxidation, with potency decreased by 13-fold and 20-fold, respectively. Conversely, reduction of the pteridine ring in the 4-(4-methylpiperazin-1-yl) derivative **18f** to give **18i** increased potency by a factor of about 2.5. In the 6,5-fused heteroaromatic compounds studied, **22** and **24** showed similar and potent inhibition of lipid peroxidation whereas compound **23** was half as potent. The role of lipophilicity (as assessed from calculated clogP values; Table 1) is not clear, but substituent bulk plays a significant role.

## *In vivo* anti-inflammatory activity

LOX has also been associated with inflammation and ulcerative colitis [45]. In the present study, a model for colitis involving intracolonic administration of aqueous 4% acetic acid in the rat was used, leading to acute inflammatory reaction [46]. Treated rats presented partial to diffuse petechial bleeding, single erosion and limited ulceration indicating an overall healing effect of the compounds. Substituted pteridines presenting a satisfactory combination of activities **5a**, **18a**, **18d** and **18f** were tested using this *in vivo* model characterized by diffuse exfoliated mucosa as well as multiple and extended erosion and ulcers of the colon (Table 3). No mortality was encountered. Pteridine **5a** (score 1–2) was the most potent in this series, followed by its 4-(*N*-benzyl) analog, **18a**. Rats treated with **5a** or **18a** showed less loss in body weight compared with the control group.

6-chloro-2-(4-methylpiperazin-1-yl)-*N*-(thiophen-2-ylmethyl)quinazolin-4-amine possesses *in vivo* anti-inflammatory properties in the rat [47], so on the basis of its close structural analogy with pteridine **18f**, that latter was tested for anti-inflammatory effects using the carrageenin paw edema model (Table 4). The incipient pattern of this edema is characterized by the effects of histamine and 5-hydroxytryptamine. After 1 h, reduction of edema in the rat paw achieved by the pteridine **18f** was appreciably greater than the reduction induced by the reference compound indomethacin, a nonselective COX-inhibitor and commonly used nonsteroidal anti-inflammatory drug. Thus, **18f** and some related pteridines offer significant protection against reactive oxygen species produced in a model of colitis, probably on account of their properties as antioxidants and radical scavengers.

## Conclusion

A general synthetic approach to *N, N,N*′-trialkylated and *N,N,N*′*N*′-tetraalkylated 2,4-diaminopteridines has been described. 2,4-diaminopteridine derivatives have been identified as a new and promising class of radical scavengers, anti-inflammatory agents and inhibitors of LOX. Potent inhibitors of soybean lipoxygenase include **9, 10a** and especially **18d** (IC_50_ = 0.1 μM). To our knowledge, 2,4-aminopteridine is a novel scaffold for LOX inhibitors, although the extent of any LOX isoform selectivity remains to be established. Many of the pteridine derivatives studied displayed potent radical-scavenging activity, especially **5c, 9, 13, 18e, 18g, 22** and **24**, of which **18g** is the most potent (IC_50_ = 0.1 μM) in the linoleic acid peroxidation assay. Several pteridine derivatives showed efficacy at 0.01 mmol/kg with little tissue damage in a rat model of colitis. 2-(4-methylpiperazin-1-yl)-*N*-(thiophen-2-ylmethyl)pteridin-4-amine (**18f**) at 0.01 mmol/kg showed 60% greater reduction of edema in rat paw than that achieved by the anti-inflammatory agent indomethacin, a nonselective COX-inhibitor. Accordingly, this study demonstrates that some pteridine derivatives have at least a dual-target action. These results prompt a more detailed structural, mechanistic and medicinal investigation of substituted 2,4-diaminopteridines, whose therapeutic potential might lead to new agents for the treatment of inflammatory bowel disease, among other inflammatory diseases.

## Future perspective

Inflammation is a multifactorial phenomenon that is implicated in a wide range of diseases. Enhanced formation of ROS by phagocytic leukocytes during the process of inflammation leads to tissue dysfunction and damage in a number of pathological conditions. ROS oxidize lipids generating peroxides and aldehydes that have pronounced biological effects including damage to DNA and protein, selective alterations in cell signaling and cytotoxicity [42]. Oxidative stress evidently plays a crucial role in those processes.

Given the importance of radical species in inflammation there is an unmet and timely need for new radical-scavenging agents. In addition, multiple-target anti-inflammatory agents have potential for the control of a range of diseases including arthritis, cancer and atherosclerosis. The wide-ranging biological activities of pteridine derivatives, including the reduction in reperfusion injury by the 4-amino analog of tetrahydrobiopterin [48], suggest that pteridines may find therapeutic applications in unexplored or little-charted areas. New pteridine derivatives could also be of value as probes of specific biological oxidation.

## Supplementary data

To view the supplementary data that accompany this paper please visit the journal website at: www.future-science.com/doi/full/10.4155/fmc.15.104

Supplementary

## Figures and Tables

**Figure 1:**
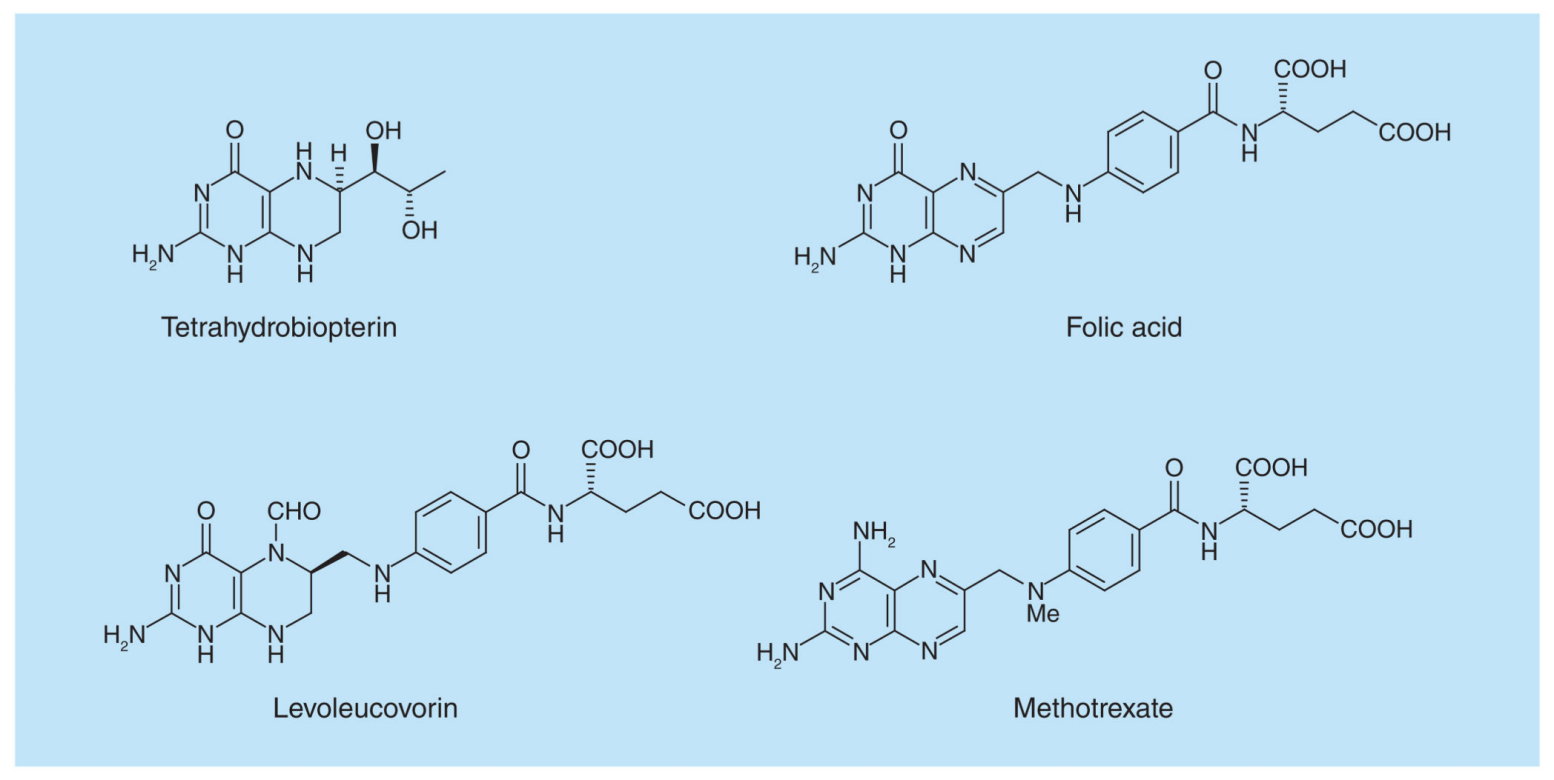
Pterin and pteridine derivatives of biological or medicinal importance.

**Table 1:** Inhibition of soybean lipoxygenase by substituted pteridines.

Entry	Compound	Structure	cLogP^43^	Lipoxygenase inhibitory activity (IC_50_ [µM] or % at 100 µM)
1	5a	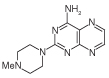	0.14	37.5 ± 0.1%
2	5b	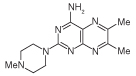	1.08	100 ± 0.3
3	5c	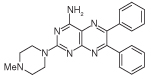	3.73	22.5 ± 0.1%
4	9	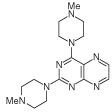	0.75	5.0 ± 0.1
5	10a	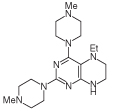	3.25	5.0 ± 0.1
6	10b	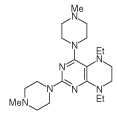	3.99	55 ± 0.2
7	13	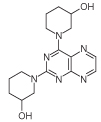	-0.16	60 ± 0.3
8	18a	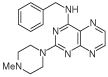	2.42	31.5 ± 0.2%
9	18b	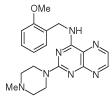	2.22	40 ± 0.2%
10	18c	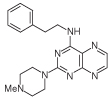	3.07	22.5 ± 0.2%
11	18d	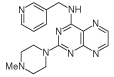	0.92	0.10 ± 0.01
12	18e	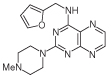	1.59	24 ± 0.3%
13	18f	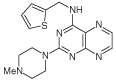	2.06	55 ± 0.8
14	18g	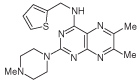	3.01	88.5 ± 1.2
15	20a	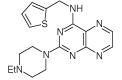	2.59	43 ± 0.7
16	20b	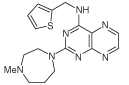	2.05	62.5 ± 0.5
17	22	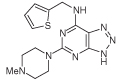	1.88	80 ± 2.1
18	23	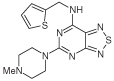	3.57	19.5 ± 0.1%
19	24	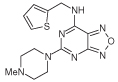	3.77	74 ± 3.5
20	NDGA	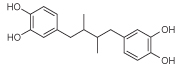	3.92	1.08 ± 0.34^40^

**Table 2:** Reducing ability in 2,2-diphenyl-1-picrylhydrazl assay, scavenging activity of hydroxyl radicals, and *in vitro* antilipid peroxidation activity of substituted pteridines.

Compound	RA (%) 100μM		Hydroxyl radicals scavenged (%)†	AAPH IC_50_ (μM)
	20 min	60 min		
5a	15	14	98	41 ± 1.1
5b	0	13	94	40 ± 0.8
5c	10	6	100	0.50 ± 0.03
9	8	14	96	0.73 ± 0.2
10a	79	81	96	15 ± 0.2
10b	98	97	98	10 ± 0.43
13	6	13	95	0.33 ± 0.01
18a	2	7	91	22 ± 0.8
18b	13	20	90	21 ± 0.9
18c	11	18	97	10 ± 0.1
18d	10	11	95	32.5 ± 1.0
18e	10	17	94	0.33 ± 0.03
18f	6	4	96	100 ± 1.5
18g	12	12	100	0.10 ± 0.01
20a	4	11	92	20 ± 0.5
20b	15	19	94	41 ± 0.3
21	4	6	94	0.29 ± 0.02
22	7	10	96	0.60 ± 0.05
23	16	18	94	0.32 ± 0.02
NDGA	81	83	–	–
Trolox	–	–	73	55.5

† Pteridine derivatives were present at 100 µM.

APH: Antilipid peroxidation; NDGA: Nordihydroguaiaretic acid; RA: Reducing ability.

**Table 3:** *In vivo* colitis studies†.

Compound	Change in body weight (%)	Score (levels 0–5)
Vehicle (control)	4.5	No activity
Vehicle + acetic acid	-6.6	Diffuse exfoliated mucosa, multiple erosion and ulcers (4–5)
Vehicle + **5a**	-1.1	One rat presented normal appearance (0); the rest exhibited hyperemia and petechial bleeding (1–2)
Vehicle + **18a**	-4.2	One rat exhibited hyperemia (1); the rest presented petechial bleeding (patchy to diffuse) (2–3)
Vehicle + **18d**	-7	One rat exhibited patchy petechial bleeding (2), and another diffuse petechial bleeding (3); the rest presented partial exfoliated mucosa or single erosion or ulcer
Vehicle + **18f**	-10.7	All rats exhibited partial to diffuse exfoliated mucosa or single/multiple erosion or ulceration (4–5)

† In each case there were two groups, each of three rats.

**Table 4:** Inhibition of carrageenin-induced rat paw edema.

Compound	Reduction of rat paw edema after 1 h (%)
**18f**	41†
Indomethacin	25†

† Mean of two experiments.

## Key terms

**Oxidative stress:** Cellular damage resulting from the action of reactive oxygen species on biological molecules in which detoxification of the reactive intermediates and/or molecular repair is insufficient.

**Inflammation:** Pattern of biological responses arising in attempts to overcome challenges by harmful stimuli, including damaged cells, irritants or pathogens.

**Reactive oxygen species:** Chemically reactive oxygen species such as anions, radical anions, radicals and peroxides formed as by-products of metabolism.

**Lipoxygenases:** Iron-containing redox enzymes that catalyze the oxygenation of fatty acids, lipoproteins and other molecules.

**Pteridines:** Bicyclic heteroaromatic compounds comprising pyrazino[2,3-*d*]pyrimidine ring fusion.

## Executive summary

- Reactive oxygen species are associated with inflammation implicated in cancer, atherosclerosis and autoimmune diseases. The pteridine ring, suitably substituted, offers a diverse array of biological activity with potential for the treatment of those diseases.
- New *N*-alkylated 2,4-diaminopteridines were prepared in good chemical yields and possess drug-like properties.
- *N*-alkylated 2,4-diaminopteridines were shown to possess a range of antioxidant properties and to be inhibitors of lipoxygenase, depending on their *N*-substitution.
- *N*-alkylated 2,4-diaminopteridines are dual-target agents, and can be radical scavengers and inhibitors of lipoxygenase. Some also exhibited potent anti-inflammatory properties, either in an *in vivo* model of colitis or in a model of odema.
