# Supplementary material for: Pteridine-2,4-diamine derivatives as radical scavengers and inhibitors of lipoxygenase that can possess anti-inflammatory properties
Source: Future Med Chem. Author manuscript; Available in PMC 2017 Jun 7. (PMC5462101; doi:10.4155/fmc.15.104)
Supplement: Supplementary [file NIHMS66069-supplement-Supplementary.doc]

## Pteridine-2,4-diamine Derivatives as Radical Scavengers and Inhibitors of Lipoxygenase that can possess Anti-Inflammatory Properties

Eleni Pontiki, Dimitra Hadjipavlou-Litina, Alexandros Patsilinakos, Trang M. Tran and Charles M. Marson

**Supporting Information**

**1. Chemistry**

**2. Experiments *in silico***

**3. Experiments *in vitro***

**4. Experiments *in vivo***

**1. Chemistry**

**General**

All chemicals were used as supplied. Solvents used were reagent grade, and anhydrous solvents were obtained from Anhydrous Engineering (USA) solvent systems after passing through an alumina column. Compound homogeneity was monitored by ascending thin-layer chromatography, performed on Merck 0.2 mm aluminum-backed silica gel 60 F_254_ plates and visualized using ultraviolet light. Flash column chromatography was performed using Merck 0.040 to 0.063 mm, 230 to 400 mesh silica gel. Evaporation refers to the removal of solvent under reduced pressure. Melting points were determined using an Electrothermal digital melting point apparatus. Infrared (IR) spectra were recorded on a Perkin-Elmer spectrum 100 FT-IR spectrometer as neat powders or as thin films. ^1^H NMR spectra were recorded at 300 MHz on a Bruker AMX300 spectrometer, at 400 MHz on a Bruker AMX400, at 500 MHz on a Bruker Avance 500 spectrometer, or at 600 MHz on a Bruker Avance 600 spectrometer in the stated solvent; chemical shifts are reported in δ (ppm) relative to the internal reference tetramethylsilane. Mass spectra were obtained on a Fisons VG70-SE mass spectrometer or Thermo Finnigan MAT900xp instrument. Purity of tested compounds was assessed to be at least 95% by HPLC-MS unless otherwise indicated.

**Synthetic methods and characterisation of the new derivatives**

The following compounds were prepared according to the literature: 4,6-diamino-2-thiopyrimidine (**1**);[22] 4,6-diamino-2-methylthiopyrimidine (**2**) [20].

**4,6-Diamino-5-nitroso-2-methylthiopyrimidine** (**3**). In a modification of a literature procedure [24]. 4,6-Diamino-2-methylthiopyrimidine (**2**) (3.19 g, 20.4 mmol) was dissolved in a mixture of water (153 mL) and glacial acetic acid (16 mL), and the mixture was cooled to 0 °C. To this mixture was added slowly a solution of sodium nitrite (3.06 g, 44.3 mmol) in water (30 mL) previously cooled to 0 °C. The turquoise mixture was stirred at 0 °C for 2 h and then left to stand at 4 °C (refrigerator) for at least a further 2 h or overnight. Filtration and washing with water (4 x 40 mL) afforded a solid that was dried at 100-120 °C for 16 h to give **3** (3.59 g, 95%) as a turquoise microprisms. A run of four times the scale gave a 70% yield.

**4,6-Diamino-2-(4-methylpiperazin-1-yl)-5-nitrosopyrimidine** (**4**). A literature procedure [23] was adapted. To the pyrimidine **3** (1.23 g, 6.64 mmol) in ethanol (6.0 mL) was added 1-methylpiperazine (3.0 mL, 27 mmol). The mixture was heated under reflux for 45 min. Water (6.0 mL) was then added and the mixture again heated under reflux for a further 40 min. The solution was immediately and rapidly cooled in ice to give small fine violet prisms. The mixture was kept at 3-4 °C for at least 3 h, preferably overnight. The precipitate was filtered and washed with plenty of water, then dried under vacuum at room temperature to give **4** (0.904 g, 57%) as fine violet prisms. Anal calc: C, 45.56, H, 6.37, N, 41.32. Found: C, 45.44, H, 6.43, N, 41.21. A run of four times the scale gave the same yield.

**2-(4-Methylpiperazin-1-yl)pteridin-4-amine (5a)**. The 5-nitrosopyrimidine **4** (2.20 g, 9.28 mmol) was heated in boiling water (25 mL) to give a purple suspension. Excess sodium dithionite was added until the solution became clear yellow. The cooled solution was evaporated to dryness. To the residue was added methanol (17.5 mL) then aqueous 40% glyoxal (1.27 mL, 11.1 mmol) followed by hydrochloric acid (2 M, 5 drops). The mixture was heated at reflux for 24 h, or until completion of the reaction as indicated by t.l.c. The yellow solution was extracted with a mixture of 2:1 chloroform:water. After further extraction with chloroform (3 x 30 mL), the combined organic layers were washed with water, dried over MgSO_4_, filtered and evaporated. The residue was recrystallized from chloroform, and the precipitate was filtered and washed with cold chloroform, then dried under vacuum to give **5a** as yellow microprisms (0.44 g, 36%), mp 210-211 °C; ν_max_ (cm^-1^) 3347, 3294, 3222 (sh), 3186, 2929, 2858, 2794, 1640, 1587; ^1^H NMR (500 MHz, CDCl_3_) δ 8.65 (1 H, d, *J*=1.0 Hz), 8.17 (1 H, d, *J*=1.0 Hz), 4.97 (4H, br s), 2.44 (4H, br s), 2.29 (3H, s); ^13^C NMR (125 MHz, CDCl_3_) δ 162.4, 160.3, 156.3, 150.8, 137.8, 123.1, 55.1, 46.2, 44.0; *m/z* (EI, %) 245 (M^+^, 100); HRMS *m/z* M^+^ calcd. for C_11_H_15_N_7_ 245.1393, found: 245.1384.

**6,7-Dimethyl**-**2-(4-methylpiperazin-1-yl)pteridin-4-amine (5b)**. The 5-nitrosopyrimidine **4** (0.174 g, 0.73 mmol) was heated in boiling water (1.5 mL) to give a purple suspension. Excess sodium dithionite was added until the solution became clear yellow. The cooled solution was evaporated to dryness. To the residue was added methanol (2.5 mL) then butane-2,3-dione (0.069 g, 0.80 mmol) followed by hydrochloric acid (2 M, 5 drops). The mixture was heated at reflux for 24 h or until completion of the reaction, as indicated by t.l.c. The mixture was worked up as described for **5a** and the product recrystallized from chloroform to give **5b** as pale yellow microprisms (0.137 g, 68%); ν_max_ (cm^-1^) 3323 (s), 2936, 2841, 2789, 1620 (s), 1580, 1541 (s); ^1^H NMR (500 MHz, CDCl_3_) δ 4.97 (4H, br s), 2.46 (4H, m), 2.61 (3H, s), 2.54 (3H, s), 2.33 (3H, s); ^13^C NMR (125 MHz, CDCl_3_) δ 161.0, 160.2, 160.1, 154.7, 146.5, 119.5, 55.2, 46.3, 44.0, 23.2, 22.0; HRMS *m/z* M^+^ calcd. for C_13_H_19_N_7_ 273.1697, found: 273.1703.

**2-(4-Methylpiperazin-1-yl)-6,7-diphenylpteridin-4-amine (5c)**. The 5-nitrosopyrimidine **4** (0.22 g, 0.93 mmol) was heated in boiling water (2.5 mL) to give a purple suspension. Excess sodium dithionite was added until the solution became clear yellow. The cooled solution was evaporated to dryness. To the residue was added methanol (2.5 mL) then benzil (0.194 g, 0.93 mmol) followed by hydrochloric acid (2 M, 5 drops). The mixture was heated at reflux for 24 h, or until completion of the reaction as indicated by t.l.c. The mixture was worked up as described for **5a** and the product recrystallized from chloroform to give **5c** as yellow microprisms (0.22 g, 59%), mp 107-109 °C; ν_max_ (cm^-1^) 3446(w), 2964(w), 2936(w), 2847(w), 2804(w), 1627, 1573(s); ^1^H NMR (500 MHz, CDCl_3_) δ 7.50 (2H, d, *J*=2.0 Hz), 7.41 (2H, d, *J*=2.0 Hz), 7.35-7.23 (6H, m), 4.04 (4H, br s), 2.49 (4H, t, *J*=5.0 Hz), 2.34 (3H, s); ^13^C NMR (125 MHz, CDCl_3_) δ 162.2, 160.6, 159.3, 154.6, 146.8, 138.8, 138.7, 130.1, 129.7, 129.1, 128.3, 128.2, 128.0, 120.6, 55.2, 46.2, 44.1; HRMS *m/z* M^+^ calcd. for C_23_H_23_N_7_ 247.0886, found: 247.0891.

**4-Amino**-**2,6-bis(4-methylpiperazin-1-yl)pyrimidine (7)**. 4-Amino-2,6-dichloropyrimidine (**6**) (1.0 g, 6.1 mmol) was added in portions with efficient stirring to 1-methylpiperazine (0.81 mL, 7.3 mmol), keeping the temperature below 50 °C. The solution was then heated at reflux under nitrogen for 18 h. After allowing the mixture to cool to room temperature, dichloromethane (10 mL) was added, followed by extraction with saturated aqueous sodium hydrogen carbonate (3 x 30 mL). The combined aqueous layers were extracted with dichloromethane (3 x 20 mL) and those extracts combined with the previous organic layer. Drying over Na_2_SO_4_, filtration and evaporation afforded a solid that was recrystallized from chloroform-hexane to give **7** as white microprisms (1.25 g, 71%), mp 117-118 °C; ν_max_ (cm^-1^) 3309, 3166, 2937, 2844, 2795, 1560 (s); ^1^H NMR (500 MHz, CDCl_3_) δ 5.07 (1H, s), 4.29 (2H, br s), 3.75 (4H, t, *J*=4.5 Hz), 3.53 (4H, t, *J*=5.0 Hz), 2.43 (8H, dt, *J*=5.0, 4.5 Hz), 2.32 (6H, s); ^13^C NMR (125 MHz, CDCl_3_) δ 164.6, 164.2, 161.7, 74.4, 55.2, 54.9, 46.4, 46.3, 44.1, 43.8; HRMS *m/z* [M-H]^+^ calcd. for C_14_H_24_N_7_ 290.2093, found: 290.2098.

**4-Amino**-**2,6-bis(4-methylpiperazin-1-yl)-5-nitrosopyrimidine (8)**. The pyrimidine **7** (0.50 g, 1.75 mmol) was dissolved in a mixture of water (12.3 mL) and glacial acetic acid (1.6 mL), and the mixture cooled to 0 °C. Sodium nitrite (0.13 g, 1.9 mmol) was dissolved in water (2.4 mL) and also cooled to 0 °C before adding slowly to the stirred solution of the pyrimidine **7**. The resulting purple solution was stirred 0 °C for 3 h. Aqueous sodium carbonate (5 M) was added slowly until pH 8 was reached, then the mixture was extracted with ethyl acetate (3 x 20 mL). The combined organic layers were dried over MgSO_4_, filtered and evaporated to give **8** as blue microprisms (0.54 g, 98%), mp 186-187 °C; ν_max_ (cm^-1^) 3240, 2925, 2851, 2795, 1604, 1527 (s); ^1^H NMR (500 MHz, CD_3_OD) δ 4.14 (4H, m), 3.91 (4H, br s), 2.55 (4H, m), 2.45 (4H, br s), 2.31 (3H, s), 2.30 (3H, s); ^13^C NMR (125 MHz, CD_3_OD) δ 163.7, 161.2, 154.0, 141.2, 56.3, 55.8, 46.0, 45.9; HRMS *m/z* [M-H]^+^ calcd. for C_14_H_23_N_8_O 319.1995, found: 319.1992.

**2,4-Bis(4-methylpiperazin-1-yl)pteridin-4-amine (9)**. The 5-nitrosopyrimidine **8** (0.40 g, 1.20 mmol) was heated in boiling water (3.2 mL) to give a suspension. Excess sodium dithionite was added until the solution became clear yellow. The cooled solution was evaporated to dryness. To the residue was added methanol (2.3 mL) then aqueous 40% glyoxal (0.17 mL, 1.4 mmol) followed by hydrochloric acid (2 M, 5 drops). The mixture was heated at reflux for 7 h, or until completion of the reaction as indicated by t.l.c. The yellow solution was extracted with a mixture of 2:1 chloroform:water. After further extraction with chloroform (3 x 30 mL), the combined organic layers were washed with water, dried over MgSO_4_, filtered and evaporated. Column chromatography of residue (5:95 methanol: chloroform) gave **9** as yellow microprisms (0.195 g, 48%), mp 121-122 °C; ν_max_ (cm^-1^) 2921, 2847, 2793, 1577 (s); ^1^H NMR (500 MHz, CDCl_3_) δ 8.56 (1 H, d, *J*=2.0 Hz), 8.16 (1 H, d, *J*=2.0 Hz), 4.34 (4H, m), 3.94 (4H, m), 2.53 (4H, t, *J*=5.1 Hz), 2.44 (4H, t, *J*=5.1 Hz), 2.32 (3H, s), 2.30 (3H, s); ^13^C NMR (125 MHz, CDCl_3_) δ 160.0, 159.5, 158.1, 149.0, 135.7, 126.0, 55.3, 55.1, 54.9, 47.4, 46.3, 46.1, 44.0, 43.7; HRMS *m/z* [M+H]^+^ calcd. for C_16_H_25_N_8_ 329.2202, found: 329.2190.

**5-Ethyl-2,4-bis(4-methylpiperazin-1-yl)-5,6,7,8-tetrahydropteridine (10a)**. Sodium triacetoxyborohydride (0.73 g, 3.4 mmol) was added to a stirred solution of pteridine **9** (0.125 g, 0.38 mmol) in glacial acetic acid (2.0 mL). The mixture was stirred for 48 h, then evaporated, and the residue was dissolved in ethyl acetate (10 mL). The solution was washed with aqueous sodium carbonate (0.1 M), dried over MgSO_4_, filtered and evaporated. Column chromatography of the residue (8:1:1 ethyl acetate:methanol:aqueous 10% ammonia) afforded **10a** as yellow microprisms (0.040 g, 29%), mp 139-140 °C; ν_max_ (cm^-1^) 3190, 2925, 2794, 1577, 1552 (s); ^1^H NMR (500 MHz, CDCl_3_) δ 4.83 (1 H, br s), 4.34 (4H, m), 3.68 (8H, m), 3.31 (2H, m), 2.99 (2H, t, *J*=5.0 Hz), 2.73 (2H, q, *J*=7.1 Hz), 2.41 (8H, m), 2.28 (6H, s), 1.04 (3H, t, *J*=7.1 Hz); ^13^C NMR (125 MHz, CDCl_3_) δ 157.1, 156.9, 156.0, 103.9, 55.6, 55.3, 45.7, 45.6, 45.4, 44.1, 44.0, 42.9, 35.6, 12.9; HRMS *m/z* M^+^ calcd. for C_18_H_32_N_8_ 360.2744, found: 360.2748.

**5,8-Diethyl-2,4-bis(4-methylpiperazin-1-yl)-5,6,7,8-tetrahydropteridine (10b)**. Sodium triacetoxyborohydride (1.28 g, 0.50 mmol) was added to a stirred solution of pteridine **9** (0.165 g, 0.50 mmol) in glacial acetic acid (2.0 mL). The mixture was stirred for 48 h, then evaporated, and the residue was dissolved in ethyl acetate (10 mL). The solution was washed with aqueous sodium carbonate (0.1 M), dried over MgSO_4_, filtered and evaporated. Column chromatography of the residue (8:1:1 ethyl acetate:methanol:aqueous 10% ammonia) afforded **10b** as a viscous brown oil (0.090 g, 46%); ν_max_ (cm^-1^) 3192, 2928, 2837, 2791, 1547 (s); ^1^H NMR (500 MHz, CDCl_3_) δ 3.64 (8H, m), 3.55 (2H, q, *J*=7.0 Hz), 3.26 (2H, m), 2.97 (2H, m), 2.70 (2H, q, *J*=7.5 Hz), 2.39 (8H, m), 2.27 (3H, s), 2.26 (3H, s), 1.07 (3H, t, *J*=7.0 Hz), 1.00 (3H, t, *J*=7.5 Hz); ^13^C NMR (125 MHz, CDCl_3_) δ 156.9, 156.8, 155.8, 104.8, 55.7, 55.6, 46.4, 45.9, 45.7, 45.6, 44.1, 43.1, 42.7, 40.4, 12.9, 11.9; HRMS *m/z* M^+^ calcd. for C_20_H_36_N_8_ 388.3057, found: 388.3057.

**1,1’-(6-Aminopyrimidine-2,4-diyl)dipiperidin-3-ol (11)**. 4-Amino-2,6-dichloropyrimidine (**6**) (0.75 g, 4.56 mmol) was added in portions with efficient stirring to (±)-3-hydroxypiperidine (1.84 g, 18.2 mmol) and *N,N*-diisopropylethylamine (1.6 mL) keeping the temperature below 50 °C. After addition, the solution was heated at reflux under nitrogen for 5 h. On standing at 25 °C, a precipitate appeared which was redissolved by addition of water. Then the mixture was partitioned between brine (30 mL) and 1:9 methanol:chloroform. After two further extractions with 1:9 methanol:chloroform, the combined organic layers were dried over MgSO_4_, filtered and evaporated. Trituration with diethyl ether afforded **11** as cream microprisms (1.20 g, 89%) mp 74-75 °C; ν_max_ (cm^-1^) 3319 (br), 2933, 2852, 1615, 1550 (s); ^1^H NMR (500 MHz, CDCl_3_) δ 5.10 (1H, s), 4.31 (2H, br s), 3.80 (3H, m), 3.73 (4H, m), 3.52 (2H, m), 2.84 (1H, m), 1.60 (4H, m), 1.50 (4H, m), 1.15 (2H, m); ^13^C NMR (125 MHz, CDCl_3_, data for both diastereoisomers) δ 164.6, 164.35, 164.3 162.1 (2 lines), 74.55, 74.4, 66.75, 66.7, 66.4, 66.35, 51.1, 51.0, 50.8, 45.2, 45.1, 44.7, 44.6 33.0, 32.9 (4 lines), 22.3, 22.2; HRMS *m/z* M^+^ calcd. for C_14_H_23_N_5_O_2_ 293.1846, found: 293.1853.

**1,1’-(6-Amino-5-nitrosopyrimidine-2,4-diyl)dipiperidin-3-ol (12)**. The pyrimidine **11** (0.70 g, 2.4 mmol) was dissolved in a mixture of water (17 mL) and glacial acetic acid (1.8 mL), and the mixture was cooled to 0 °C. Sodium nitrite (0.18 g, 2.6 mmol) was dissolved in water (3.4 mL) and also cooled to 0 °C before adding slowly to the stirred solution of the pyrimidine. The resulting purple solution was stirred 0 °C for 3 h. Aqueous sodium carbonate (5 M) was added slowly until pH 8 was reached, then the mixture was extracted with ethyl acetate (3 x 20 mL). The combined organic layers were dried over MgSO_4_, filtered and evaporated to give **12** as blue microprisms (0.55 g, 71%), mp 106-107 °C; ν_max_ (cm^-1^) 3323 (br), 2933, 2853, 1615, 1535 (s); ^1^H NMR (500 MHz, CD_3_OD) δ 4.85 (4H, br s), 4.61-4.32 (4H, m), 3.76 (1H, m) 3.70-3.17 (5H, m), 1.62-1.40 (8H, m); ^13^C NMR (125 MHz, CD_3_OD) δ 163.9, 161.6, 154.4, 140.7, 67.75, 67.5, 52.1, 51.9, 45.6, 45.5, 34.0, 24.4, 24.0; HRMS *m/z* M^+^ calcd. for C_14_H_22_N_6_O_3_ 322.1748, found: 322.1741.

**1,1’-(Pteridine-2,4-diyl)dipiperidin-3-ol (13)**. The 5-nitrosopyrimidine **12** (0.51 g, 1.54 mmol) was heated in boiling water (4.1 mL) to give a suspension. A few drops of ethanol were added followed by excess sodium dithionite until the solution became clear yellow. The cooled solution was evaporated to dryness. To the residue was added methanol (3.0 mL) then aqueous 40% glyoxal (0.22 mL, 1.75 mmol) followed by hydrochloric acid (2 M, 5 drops). The mixture was heated at reflux for 6 h, or until completion of the reaction as indicated by t.l.c. The yellow solution was extracted with a mixture of 2:1 chloroform:water. After further extraction with chloroform (3 x 30 mL), the combined organic layers were washed with water, dried over MgSO_4_, filtered and evaporated to give **13** as yellow microprisms (0.21 g, 41%), mp 139-140 °C; ν_max_ (cm^-1^) 3319 (br), 2933, 2853, 1615, 1544 (s); ^1^H NMR (500 MHz, CDCl_3_) δ 8.54 (1H, br s), 8.13 (1H, br s), 4.40-3.30 (10H, m), 2.00-1.20 (8H, m); ^13^C NMR (125 MHz, CDCl_3_) δ 161.0, 160.8, 159.8, 158.0, 149.1, 135.6, 135.5, 125.5, 66.6, 66.5, 54.1, 51.1, 48.7, 44.6, 32.65, 32.5, 32.45, 29.8, 22.6; HRMS *m/z* [M+H]^+^ calcd. for C_16_H_22_N_6_O_2_ 331.1883, found: 331.1879.

***N^4^*-Benzyl-2-methylthiopyrimidine-4,6-diamine (15a)**. The pyrimidine **14** (1.42 g, 8.1 mmol) and benzylamine (1.85 mL, 17.0 mmol) were heated at reflux in diglyme (4.0 mL) for 5 h. The cooled solution was filtered, and diethyl ether (10 mL) was added to the filtrate. The organic layer was washed with water (2 x 5 mL), dried over MgSO_4_, filtered and evaporated. Column chromatography of the residue (1:1 ethyl acetate:60-80 °C petroleum ether) afforded **15a** as white microprisms (1.03 g, 51%), mp 117-118 °C, lit. [28] mp 117-119 °C; ν_max_ (cm^-1^) 3422, 3292, 3134, 2919 (w), 2859 (w), 1636, 1582 (s); ^1^H NMR (500 MHz, CDCl_3_) δ 7.35-7.26 (5H, m), 5.16 (1H, br s), 5.11 (1H, s), 4.55 (2H, br s), 4.43 (1H, s), 4.42 (1H, s), 2.45 (3H, s); ^13^C NMR (125 MHz, CDCl_3_) δ 170.9, 163.1, 163.05, 138.4, 128.8, 127.5, 127.3, 78.8, 45.7, 13.9.

***N^4^*-(2-Methoxybenzyl)-2-methylthiopyrimidine-4,6-diamine (15b)**. The pyrimidine **14** (1.42 g, 8.1 mmol) and *o*-anisidine (2.22 mL, 17.0 mmol) were heated at reflux in diglyme (4.0 mL) for 16 h. The cooled solution was filtered, and diethyl ether (15 mL) was added to the filtrate. The organic layer was washed with water (2 x 5 mL), dried over MgSO_4_, filtered and evaporated. Column chromatography of the residue (1:1 ethyl acetate:60-80 °C petroleum ether) afforded **15b** as beige prisms (1.40 g, 63%), mp 68-69 °C; ν_max_ (cm^-1^) 3420, 3291, 3135, 1635, 1545 (s); ^1^H NMR (500 MHz, CDCl_3_) δ 7.26-7.23 (2H, m), 6.92-6.86 (2H, m), 5.16 (1H, br s), 5.12 (1H, s), 4.59 (2H, br s), 4.40 (1H, s), 4.39 (1H, s), 3.84 (3H, s), 2.45 (3H, s); ^13^C NMR (125 MHz, CDCl_3_) δ 170.6, 163.2, 163.1, 157.3, 128.7, 126.3, 120.6, 110.3, 78.6, 55.4, 41.1, 13.9; HRMS *m/z* M^+^ calcd. for C_13_H_16_N_4_OS 276.1039, found: 276.1046.

**2-(Methylthio)-*N*-(pyridin-3-ylmethyl)pyrimidine-4,6-diamine (15d)**. The pyrimidine **14** (1.42 g, 8.1 mmol) and 3-picolylamine (1.73 mL, 17.0 mmol) were heated at reflux in diglyme (4.0 mL) for 16 h. The mixture was dissolved in methanol and subjected to column chromatography (1:9 methanol:chloroform) to give **15d** as white microprisms (0.76 g, 38%), mp 195-196 °C; ν_max_ (cm^-1^) 3249 (br), 3073 (br), 2928, 1654, 1565 (s); ^1^H NMR (500 MHz, DMSO-*d*_6_) δ 8.77 (1H, s), 8.69 (1H, d, *J*=3.4 Hz), 7.93 (1H, d, *J*=7.0 Hz), 7.58 (1H, m), 7.50 (1H, br s), 6.40 (1H, br s), 5.43 (1H, s), 4.70 (2H, br s), 2.50 (3H, s); ^13^C NMR (125 MHz, DMSO-*d*_6_) δ 169.1, 163.5, 162.5, 149.0, 148.2, 136.0, 135.1, 123.7, 78.9, 41.7, 13.2; HRMS *m/z* M^+^ calcd. for C_11_H_13_N_5_S 247.0886, found: 247.0891.

***N*-(Furan-2-ylmethyl)-2-(methylthio)pyrimidine-4,6-diamine (15e)**. 4-The pyrimidine **14** (1.42 g, 8.1 mmol) and furfurylamine (1.5 mL, 8.5 mmol) were heated at reflux in diglyme (4.0 mL) for 7 h. The cooled solution was filtered, and diethyl ether (10 mL) was added to the filtrate. The organic layer was washed with water (2 x 5 mL), dried over MgSO_4_, filtered and evaporated. Column chromatography of the residue (1:1 ethyl acetate:60-80 °C petroleum ether) afforded **15e** as beige microprisms (0.66 g, 35%), mp 95-96 °C; ν_max_ (cm^-1^) 3417, 3299, 3145, 2925 (w), 1637, 1585 (s); ^1^H NMR (500 MHz, CDCl_3_) δ 7.33 (1H, dd, *J*=1.8, 0.8 Hz), 6.30 (1H, dd, *J*=3.3, 1.8 Hz), 6.20 (1H, dd, *J*=3.3, 0.8 Hz), 5.18 (2H, br s), 4.70 (2H, br s), 4.42 (1H, s), 4.41 (1H, s), 2.44 (3H, s); ^13^C NMR (125 MHz, CDCl_3_) δ 173.4, 165.8, 165.4, 154.5, 144.8, 113.2, 109.9, 81.7, 41.5, 16.5; HRMS *m/z* M^+^ calcd. for C_10_H_12_N_4_OS 236.0727, found: 236.0731.

**2-(Methylthio)-*N*-(thiophen-2-ylmethyl)pyrimidine-4,6-diamine (15f)**. The pyrimidine **14** (1.42 g, 8.1 mmol) and 2-thiophenemethylamine (1.74 mL, 17.0 mmol) were heated at reflux in diglyme (4.0 mL) for 11 h. The cooled solution was filtered, and diethyl ether (10 mL) was added to the filtrate. The organic layer was washed with water (2 x 5 mL), dried over MgSO_4_, filtered and evaporated. Column chromatography of the residue (1:1 ethyl acetate:60-80 °C petroleum ether) afforded **15f** as beige microprisms (1.33 g, 41%), mp 123-124 °C; ν_max_ (cm^-1^) 3465, 3378, 3298, 3159, 2928, 1626, 1569 (s); ^1^H NMR (500 MHz, CDCl_3_) δ 7.20 (1H, dd, *J*=5.0, 1.2 Hz), 6.97 (1H, dq, *J*=3.4, 1.2 Hz), 6.94 (1H, dd, *J*=5.0, 3.4 Hz), 5.16 (2H, br s), 4.63 (4H, m), 2.46 (3H, s); ^13^C NMR (125 MHz, CDCl_3_) δ 170.9, 163.0, 162.6, 141.8, 126.9, 125.4, 125.0, 79.2, 40.7, 13.9; HRMS *m/z* M^+^ calcd. for C_10_H_12_N_4_S_2_ 252.0498, found: 252.0501.

***N^4^*-Benzyl-2-methylthio-5-nitrosopyrimidine-4,6-diamine (16a)**. The pyrimidine **15a** (0.50 g, 2.0 mmol) was dissolved in a mixture of water (15 mL) and glacial acetic acid (1.6 mL), and the mixture was cooled to 0 °C. Sodium nitrite (0.30 g, 4.4 mmol) was dissolved in water (3.0 mL) and also cooled to 0 °C before adding slowly to the stirred solution of the pyrimidine. The resulting turquoise solution was stirred 0 °C for 2 h, then left to stand at 4 °C for 16 h. Filtration and drying afforded **16a** as turquoise prisms (0.52 g, 95%), mp 185-186 °C; ν_max_ (cm^-1^) 3328, 3264, 3060, 3033, 2924, 1637, 1581 (s); ^1^H NMR (500 MHz, DMSO-*d*_6_) rotamer A: δ 11.71 (1H, br s), 9.01 (1H, d, *J*=6.0 Hz), 8.09 (1H, br s), 7.40-7.22 (5H, m), 4.75 (2H, d, *J*=6.0 Hz), 2.41 (3H, s); rotamer A: δ 10.24 (1H, br s), 10.12 (1H, br s), 8.48 (1H, br s), 7.40-7.22 (5H, m), 4.75 (2H, d, *J*=3.0 Hz), 2.41 (3H, s); ^13^C NMR (125 MHz, DMSO-*d*_6_) δ 179.0, 178.6, 164.2, 161.2, 145.8, 145.0, 139.1, 138.8, 138.2, 138.0, 128.8, 128.5, 128.3, 127.6, 127.5, 127.2, 126.9, 43.8, 42.7, 13.8; HRMS *m/z* [M+H]^+^ calcd. for C_12_H_14_N_5_OS 276.0919, found: 276.0922.

***N^4^*-(2-Methoxybenzyl)-2-methylthio-5-nitrosopyrimidine-4,6-diamine (16b)**. The pyrimidine **15b** (0.95 g, 3.4 mmol) was dissolved in a mixture of water (8.5 mL) and glacial acetic acid (17 mL), and the mixture was cooled to 0 °C. Sodium nitrite (0.52 g, 7.5 mmol) was dissolved in water (5.1 mL) and also cooled to 0 °C before adding slowly to the stirred solution of **15b**. The resulting turquoise solution was stirred 0 °C for 3 h, then left to stand at 4 °C for 16 h. Filtration and drying afforded **16b** as turquoise prisms (1.0 g, 95%), mp 180-181 °C; ν_max_ (cm^-1^) 3323(br), 2933, 2853, 1543(s); ^1^H NMR (500 MHz, CDCl_3_) rotamer A: δ 11.70 (1H, br s), 7.58 (1H, br s), 7.37-7.22 (2H, m), 7.00-6.91 (2H, m), 5.88 (1H, br s), 4.73 (2H, br s), 3.96 (3H, s), 2.55 (3H, s); rotamer B: δ 10.40 (1H, br s), 8.43 (1H, br s), 7.37-7.22 (2H, m), 7.00-6.91 (2H, m), 6.01 (1H, br s), 4.89 (2H, m), 3.87 (3H, s), 2.59 (3H, s); ^13^C NMR (125 MHz, CDCl_3_) δ 180.9, 180.6, 164.5, 161.1, 157.9, 157.7, 146.3, 145.5, 138.6, 138.1, 131.1, 129.8, 129.5, 129.4, 125.4, 124.9, 120.7, 120.6, 110.6, 110.5, 55.3, 41.0, 39.6, 14.7, 14.6; HRMS *m/z* M^+^ calcd. for C_13_H_15_N_5_O_2_S 305.0941, found: 305.0942.

**2-(Methylthio)-5-nitroso-*N*-phenethylpyrimidine-4,6-diamine (16c)**. The pyrimidine **15c** (0.57 g, 2.2 mmol) was dissolved in a mixture of water (6.0 mL) and glacial acetic acid (7.0 mL), and the mixture was cooled to 0 °C. Sodium nitrite (0.39 g, 4.9 mmol) was dissolved in water (3.0 mL) and also cooled to 0 °C before adding slowly to the stirred solution of the pyrimidine. The resulting turquoise solution was stirred 0 °C for 3 h, then left to stand at 4 °C for 16 h. Filtration and drying afforded **16c** as turquoise microprisms (0.50 g, 78%), mp 179-180 °C; ν_max_ (cm^-1^) 3460, 3412, 3287, 3145, 3023 (w), 2926, 1634, 1594 (s), 1563 (s); ^1^H NMR (500 MHz, CDCl_3_) rotamer A: δ 11.43 (1H, br s), 7.65 (1H, br s), 7.34-7.30 (2H, m), 7.26-7.20 (3H, m), 6.19 (1H, br s), 3.77 (2H, dt, *J*=7.0, 6.3 Hz), 2.92 (2H, t, *J*=7.3 Hz), 2.53 (3H, s); rotamer B: δ 10.37 (1H, br s), 8.02 (1H, br s), 7.34-7.30 (2H, m), 7.26-7.20 (3H, m), 6.29 (1H, br s), 3.96 (2H, dt, *J*=7.3, 6.3 Hz), 3.03 (2H, t, *J*=7.3 Hz), 2.53 (3H, s); ^13^C NMR (125 MHz, CDCl_3_) δ 180.9, 180.7, 164.4, 161.4, 146.3, 145.9, 138.4, 138.3, 137.9, 128.9, 128.85, 128.8, 128.7, 126.9, 42.5, 41.4, 35.8, 14.7, 14.6; HRMS *m/z* [M+H]^+^ calcd. for C_13_H_16_N_5_OS 290.1076, found: 290.1076.

**2-(Methylthio)-5-nitroso-*N*-(pyridin-3-ylmethyl)pyrimidine-4,6-diamine (16d)**. The pyrimidine **15d** (0.50 g, 2.0 mmol) was dissolved in a mixture of water (7 mL) and glacial acetic acid (6.0 mL), and the mixture was cooled to 0 °C. Sodium nitrite (0.31 g, 4.5 mmol) was dissolved in water (3.0 mL) and also cooled to 0 °C before adding slowly to the stirred solution of the pyrimidine. The resulting turquoise mixture was stirred at 0 °C for 3 h, then left to stand at 4 °C for 16 h. Filtration and drying under vacuum afforded **16d** as turquoise microprisms (0.33 g, 59%), mp 180-181 °C; ν_max_ (cm^-1^) 3324 (br), 3085 (w), 2930, 2855 (w), 1577, 1547 (s); ^1^H NMR (500 MHz, DMSO-*d*_6_) rotamer A: δ 11.63 (1H, t, *J*=5.1 Hz), 9.09 (1H, s), 8.70-8.55 (2H, m), 8.13 (1H, s), 8.21 (1H, d, *J*=8.0 Hz), 7.72 (1H, dd, *J*=8.0, 5.2 Hz), 4.85 (2H, d, *J*=5.1 Hz), 2.42 (3H, s); rotamer B: δ 10.20 (1H, m), 8.80 (1H, s), 8.70-8.55 (3H, m), 8.06 (1H, d, *J*=8.0 Hz), 7.65 (1H, dd, *J*=8.0, 5.2 Hz), 4.72 (2H, d, *J*=5.1 Hz), 2.39 (3H, s); ^13^C NMR (125 MHz, DMSO-*d*_6_) δ 178.9, 178.7, 164.3, 161.5, 145.6, 145.5, 145.2, 145.0, 144.8, 144.7, 140.1, 139.9, 139.8, 138.3, 136.9, 136.1, 125.3, 125.1, 41.3, 13.9, 13.8; HRMS *m/z* [M+H]^+^ calcd. for C_11_H_13_N_6_OS 277.0872, found: 277.0872.

***N*-(Furan-2-ylmethyl)-2-(methylthio)-5-nitrosopyrimidine-4,6-diamine (16e)**. The pyrimidine **15e** (0.472 g, 2.0 mmol) was dissolved in a mixture of water (12 mL) and glacial acetic acid (4.0 mL), and the mixture was cooled to 0 °C. Sodium nitrite (0.30 g, 4.4 mmol) was dissolved in water (3.0 mL) and also cooled to 0 °C before adding slowly to the stirred solution of the pyrimidine. The resulting turquoise mixture was stirred 0 °C for 2 h, then left to stand at 4 °C for 16 h. Filtration and drying under vacuum afforded **16e** as turquoise microprisms (0.50 g, 95%), mp 157-158 °C; ν_max_ (cm^-1^) 3304, 3148, 1701, 1643, 1585 (s); ^1^H NMR (500 MHz, DMSO-*d*_6_) rotamer A: δ 11.53 (1H, br s), 9.04 (1H, br s), 8.11 (1H, br s), 7.58 (1H, m), 6.39 (1H, m), 6.30 (1H, m), 4.67 (2H, d, *J*=6.0 Hz), 2.46 (3H, s); rotamer B: δ 10.20 (1H, br s), 9.92 (1H, br s), 8.11 (1H, br s), 7.58 (1H, m), 6.39 (1H, m), 6.30 (1H, m), 4.74 (2H, d, *J*=5.5 Hz), 2.48 (3H, s); ^13^C NMR (125 MHz, DMSO-*d*_6_) δ 179.1, 178.6, 172.0, 164.1, 161.2, 151.8, 150.6, 145.7, 144.8, 142.6, 142.0, 138.9, 138.2, 110.6, 107.8, 107.3, 37.1, 35.9, 21.1, 13.8; HRMS *m/z* M^+^ calcd. for C_10_H_11_N_5_O_2_S 265.0628, found: 265.0622.

**2-(Methylthio)-5-nitroso-*N*-(thiophen-2-ylmethyl)pyrimidine-4,6-diamine (16f)**. The pyrimidine **15f** (0.50 g, 2.0 mmol) was dissolved in a mixture of water (15 mL) and glacial acetic acid (1.6 mL), and the mixture was cooled to 0 °C. Sodium nitrite (0.30 g, 4.4 mmol) was dissolved in water (3.0 mL) and also cooled to 0 °C before adding slowly to the stirred solution of the pyrimidine. The resulting turquoise solution was stirred at 0 °C for 2 h, then left to stand at 4 °C for 16 h. Filtration and drying afforded **16f** as turquoise microprisms (0.53 g, 95%), mp 186-187 °C; ν_max_ (cm^-1^) 3434, 3276, 3106, 1640, 1590 (s); ^1^H NMR (500 MHz, DMSO-*d*_6_) rotamer A: δ 11.59 (1H, br s), 9.00 (1H, br s), 8.09 (1H, br s), 7.61-7.37 (1H, m), 7.10 (1H, m), 6.95 (1H, m), 4.80 (1H, s), 4.79 (1H, s), 2.49 (3H, s); rotamer B: δ 10.20 (1H, br s), 10.11 (1H, br s), 7.62 (1H, br s), 7.50 (1H, m), 7.20 (1H, m), 6.95 (1H, m), 4.89 (2H, br s), 2.50 (3H, s); ^13^C NMR (125 MHz, DMSO-*d*_6_) δ 178.9, 178.6, 160.9, 145.7, 144.5, 141.5, 140.3, 138.8, 138.2, 128.2, 128.0, 126.8, 126.6, 126.5, 126.3, 125.9, 37.5, 23.5, 13.9, 13.7; HRMS calcd. for HRMS *m/z* [M-H]^+^ calcd. for C_10_H_12_N_5_OS_2_ 280.0327, found: 280.0329.

***N^4^*-Benzyl-2-(4-methylpiperazin-1-yl)-5-nitrosopyrimidine-4,6-diamine (17a)**. To nitrosopyrimidine **16a** (0.45 g, 1.63 mmol) in ethanol (1.5 mL) was added 1-methylpiperazine (0.72 mL, 6.5 mmol); the mixture was heated at reflux for 2 h. Water (1.5 mL) was then added and the mixture again heated under reflux for a further 1 h. The solution was immediately and rapidly cooled in ice to give small reddish-violet prisms. After standing overnight at 4 °C, filtration, washing with water and drying under vacuum afforded **17a** as a small reddish-violet prisms (0.245 g, 46%), mp 176-177 °C; ν_max_ (cm^-1^) 3234, 2933, 2852, 2788, 1567 (s); ^1^H NMR (500 MHz, DMSO-*d*_6_) rotamer A: δ 11.73 (1H, br s), 8.26 (1H, br s), 7.49 (1H, br s), 7.38-7.19 (5H, m), 4.57 (1H, s), 4.56 (1H, s), 3.81 (4H, m), 2.30 (4H, m), 2.17 (3H, s); rotamer B: δ 10.35 (1H, br s), 9.39 (1H, br s), 7.88 (1H, br s), 7.38-7.19 (5H, m), 4.64 (1H, s), 4.63 (1H, s), 3.81 (4H, m), 2.48 (4H, m), 2.16 (3H, s); ^13^C NMR (125 MHz, DMSO-*d*_6_) δ 165.3, 162.3, 160.6, 160.4, 150.3, 149.5, 139.7, 139.5, 137.1, 136.5, 128.4, 128.3, 128.2, 127.7, 127.6, 127.4, 127.1, 126.7, 54.4, 45.6, 43.7, 43.6, 43.55, 43.5, 42.5; HRMS *m/z* [M-H]^+^ calcd. for C_16_H_20_N_7_O 326.1729, found: 326.1734.

***N*-(2-Methoxybenzyl)-2-(4-methylpiperazin-1-yl)-5-nitrosopyrimidine-4,6-diamine (17b)**. To nitrosopyrimidine **16b** (0.66 g, 2.1 mmol) in ethanol (2.0 mL) was added 1-methylpiperazine (0.96 mL, 8.7 mmol). The mixture was heated at reflux for 2 h. Water (2.0 mL) was then added and the mixture again heated under reflux for a further 1 h. The solution was evaporated and purified by column chromatography (5:95 methanol:chloroform) to give **17b** as dark red microprisms (0.405 g, 42%), mp 88-89 °C; ν_max_ (cm^-1^) 3308 (br), 2936, 2851, 2792, 1568 (s), 1527 (s, br); ^1^H NMR (500 MHz, CDCl_3_) rotamer A: δ 11.60 (1H, br s), 7.58 (1H, br s), 7.31-7.22 (2H, m), 6.91-6.86 (2H, m), 5.50 (1H, br s), 4.62 (2H, t, *J*=6.0 Hz), 4.10-3.85 (4H, m), 3.83 (3H, s), 2.51-2.31 (4H, m), 2.31 (3H, s); rotamer B: δ 10.60 (1H, br s), 7.96 (1H, br s), 7.32-7.22 (2H, m), 7.00-6.91 (2H, m), 6.05 (1H, br s), 4.75 (2H, t, *J*=5.5 Hz), 4.10-3.85 (4H, m), 3.84 (3H, s), 2.51-2.31 (4H, m), 2.31 (3H, s); ^13^C NMR (125 MHz, DMSO-*d*_6_) δ 165.8, 162.5, 161.2, 157.58, 157.7, 150.9, 150.1, 137.1, 136.8, 129.8, 129.4, 129.1, 128.9, 126.1, 125.8, 120.5, 120.4, 110.5, 110.4, 110.3, 55.5, 55.4, 55.2, 55.1, 55.0, 54.9, 46.1, 44.6, 44.3, 44.2, 44.1, 40.4, 38.9; HRMS *m/z* [M+H]^+^ calcd. for C_17_H_24_N_7_O_2_ 358.1992, found: 358.1989.

**2-(4-Methylpiperazin-1-yl)-5-nitroso-*N*-phenethylpyrimidine-4,6-diamine (17c)**. To nitrosopyrimidine **16c** (0.61 g, 2.1 mmol) in ethanol (2.0 mL) was added 1-methylpiperazine (0.96 mL, 1.1 mmol). The mixture was heated at reflux for 2 h. Water (2.0 mL) was then added and the mixture again heated under reflux for a further 1 h. The solution was evaporated and the residue was purified by column chromatography (15:95 methanol:ethyl acetate) to give **17c** as reddish-violet microprisms (0.48 g, 67%), mp 162-163 °C; ν_max_ (cm^-1^) 3225 (br), 2935, 2846 (w), 2796, 1568 (s), 1520 (s); ^1^H NMR (500 MHz, CDCl_3_) rotamer A: δ 11.40 (1H, br s), 7.35-7.15 (6H, m), 5.30 (1H, br s), 4.03-3.90 (4H, m), 3.66 (2H, m), 2.89 (2H, t, *J*=7.5 Hz), 2.48-2.42 (4H, m), 2.33 (3H, s); rotamer B: δ 10.57 (1H, br s), 7.59 (1H, t, *J*=5.0 Hz), 7.35-7.15 (5H, m), 5.51 (1H, d, *J*=5.0 Hz), 4.03-3.90 (4H, m), 3.81 (2H, m), 2.97 (2H, t, *J*=7.5 Hz), 2.48-2.42 (4H, m), 2.34 (3H, s); ^13^C NMR (125 MHz, CDCl_3_) δ 165.7, 162.7, 161.9, 161.2, 150.8, 150.4, 138.9, 138.8, 136.9, 136.6, 128.8, 128.75, 128.7, 126.7, 126.6, 55.5, 55.1, 55.0, 54.9, 46.1, 44.3, 44.2, 44.15, 44.1, 42.2, 41.3, 35.8, 35.5; HRMS *m/z* [M+H]^+^ calcd. for C_17_H_24_N_7_O 342.2042, found: 342.2046.

**2-(4-Methylpiperazin-1-yl)-5-nitroso-*N*-(pyridin-3-ylmethyl)pyrimidine-4,6-diamine (17d)**. To nitrosopyrimidine **16d** (0.607 g, 2.1 mmol) in ethanol (2.0 mL) was added 1-methylpiperazine (0.96 mL, 8.7 mmol). The mixture was heated at reflux for 2 h. Water (2.0 mL) was then added and the mixture again heated under reflux for a further 1 h. The solution was evaporated and the residue purified by column chromatography (5:95 methanol:chloroform) to give **17d** as dark red microprisms (0.48 g, 67%), mp 162-163 °C; ν_max_ (cm^-1^) 3293 (br), 3176 (br), 2934, 2858 (w), 2796, 1635, 1568 (s); ^1^H NMR (500 MHz, CDCl_3_) rotamer A: δ 11.69 (1H, br s), 8.49 (1H, d, *J*=4.6 Hz), 7.59 (1H, d, *J*=7.9 Hz), 7.24 (2H, m), 5.36 (1H, m), 4.62 (2H, d, *J*=5.5 Hz), 3.94-3.88 (4H, m), 2.57-2.30 (4H, m), 2.28 (3H, s); rotamer B: δ 10.48 (1H, br s), 8.63 (1H, s), 8.52 (1H, d, *J*=4.5 Hz), 7.93 (1H, t, *J*=6.0 Hz), 7.68 (1H, d, *J*=7.8 Hz), 7.24 (1H, m), 6.05 (1H, m), 4.77 (2H, d, *J*=6.0 Hz), 3.94-3.88 (4H, m), 2.57-2.30 (4H, m), 2.28 (3H, s); ^13^C NMR (125 MHz, CDCl_3_) δ 165.6, 162.9, 161.1, 161.0, 150.7, 149.4, 149.0, 148.9, 137.0, 136.6, 135.3, 134.0, 133.6, 123.6, 122.9, 55.0, 54.9, 54.8, 46.0, 44.4, 44.3, 44.2, 42.2, 41.0; HRMS *m/z* [M+H]^+^ calcd. for C_15_H_21_N_8_O 329.1838, found: 329.1842.

***N*-(Furan-2-ylmethyl)-2-(4-methylpiperazin-1-yl)-5-nitrosopyrimidine-4,6-diamine (17e)**. To the nitrosopyrimidine **16e** (0.432 g, 1.63 mmol) in ethanol (1.5 mL) was added 1-methylpiperazine (0.72 mL, 6.5 mmol). The mixture was heated at reflux for 2 h. Water (1.5 mL) was then added and the mixture again heated under reflux for a further 1 h. The solution was immediately and rapidly cooled in ice to give small dark red prisms. (If no crystals are formed the solution is evaporated and the residue recrystallised from aqueous ethanol). After standing overnight at 4 °C, filtration, washing with water and drying under vacuum afforded **17e** as a small reddish-violet prisms (0.284 g, 55%), mp 168-169 °C; ν_max_ (cm^-1^) 3221, 3124, 2950, 2919, 2853, 2796, 1603, 1569 (s); ^1^H NMR (500 MHz, DMSO-*d*_6_) rotamer A: δ 11.58 (1H, br s), 8.29 (1H, br s), 7.83 (1H, br s), 7.53 (1H, m), 6.39 (1H, m), 6.28 (1H, m), 4.58 (2H, d, *J*=5.0 Hz), 3.90-3.75 (4H, m), 2.35-2.23 (4H, m), 2.18 (3H, s); rotamer B: δ 10.32 (1H, br s), 9.16 (1H, br s), 7.91 (1H, br s), 7.53 (1H, m), 6.39 (1H, m), 6.28 (1H, m), 4.63 (2H, d, *J*=4.5 Hz), 3.90-3.75 (4H, m), 2.35-2.23 (4H, m), 2.18 (3H, s); ^13^C NMR (125 MHz, DMSO-*d*_6_) δ 165.3, 162.4, 160.5, 160.3, 152.4, 151.1, 150.2, 149.3, 142.5, 141.8, 137.2, 136.5, 110.6, 110.5, 107.5, 106.9, 54.4, 43.7, 43.6, 43.5, 43.4, 40.1, 39.0, 36.9, 35.6; HRMS *m/z* M^+^ calcd. for C_14_H_19_N_7_O_2_ 317.1595, found: 317.1603.

**2-(4-Methylpiperazin-1-yl)-5-nitroso-*N*-(thiophen-2-ylmethyl)-pyrimidine-4,6-diamine (17f)**. To nitrosopyrimidine **16f** (0.458 g, 1.63 mmol) in ethanol (1.5 mL) was added 1-methylpiperazine (0.72 mL, 6.5 mmol). The mixture was heated at reflux for 2 h. Water (1.5 mL) was then added and the mixture again heated under reflux for a further 1 h. The solution was immediately and rapidly cooled in ice to give small reddish-violet prisms. (If no crystals are formed the solution is evaporated and the residue recrystallised from aqueous ethanol). After standing overnight at 4 °C, filtration, washing with water and drying under vacuum afforded **17f** as a small reddish-violet prisms (0.227 g, 42%), mp 191-192 °C; ν_max_ (cm^-1^) 3328, 2934, 2789, 1607, 1569 (s); ^1^H NMR (500 MHz, DMSO-*d*_6_) rotamer A: δ 11.61 (1H, br s), 8.29 (1H, s), 7.52 (1H, s), 7.37 (1H, dd, *J*=5.1, 1.0 Hz), 7.03 (1H, m), 6.93 (1H, dd, *J*=5.1, 3.5 Hz), 4.73 (2H, d, *J*=6.0 Hz), 3.96-3.82 (4H, m), 2.50-2.30 (4H, m), 2.22 (3H, s); rotamer B: δ 10.32 (1H, d, *J*=4.5 Hz), 9.38 (1H, t, *J*=6.2 Hz), 7.91 (1H, d, *J*=4.5 Hz), 7.37 (1H, dd, *J*=5.1, 1.0 Hz), 7.03 (1H, m), 6.93 (1H, dd, *J*=5.1, 3.5 Hz), 4.79 (2H, d, *J*=6.2 Hz), 3.96-3.82 (4H, m), 2.50-2.30 (4H, m), 2.18 (3H, s); ^13^C NMR (125 MHz, DMSO-*d*_6_) δ 165.3, 162.1, 160.4, 160.2, 150.2, 149.0, 142.2, 141.0, 137.1, 136.5, 126.5, 126.4, 126.3, 126.0, 125.7, 125.2, 54.6, 54.45, 54.44, 45.6, 43.8, 43.7, 43.55, 43.5, 38.4, 37.2; HRMS *m/z* [M-H]^+^ calcd. for C_14_H_18_N_7_OS 332.1294, found: 332.1294.

***N*-Benzyl-2-(4-methylpiperazin-1-yl)pteridin-4-amine (18a)**. The 5-nitrosopyrimidine **17a** (0.40 g, 1.2 mmol) was heated in boiling water (3.2 mL) to give a suspension. Excess sodium dithionite was then added until the solution became clear yellow. The cooled solution was evaporated to dryness. To the residue was added methanol (2.3 mL) then aqueous 40% glyoxal (0.17 mL, 1.4 mmol) followed by hydrochloric acid (2 M, 5 drops). The mixture was heated at reflux for 7 h, or until completion of the reaction as indicated by t.l.c. The yellow solution was extracted with a mixture of 2:1 chloroform:water. After further extraction with chloroform (3 x 30 mL), the combined organic layers were washed with water, dried over MgSO_4_, filtered and evaporated. Column chromatography of the residue (5:95 methanol:chloroform) afforded **18a** as yellow microprisms (0.195 g, 48%), mp 163-164 °C; ν_max_ (cm^-1^) 3261, 2929, 2788, 1598(s), 1571 (s); ^1^H NMR (500 MHz, CDCl_3_) δ 8.61 (1H, d, *J*=2.0 Hz), 8.09 (1H, d, *J*=2.0 Hz), 7.35-7.25 (5H, m), 7.19 (1H, t, *J*=5.9 Hz), 4.74 (2H, d, *J*=5.9 Hz), 3.99 (4H, br s), 2.42 (4H, m), 2.29 (3H, s); ^13^C NMR (125 MHz, CDCl_3_) δ 160.4 160.1, 156.3, 150.3, 137.2, 128.8, 128.6, 128.0, 127.9, 123.6, 55.1, 46.3, 44.9, 44.1; HRMS *m/z* [M+H]^+^ calcd. for C_18_H_22_N_7_ 336.1934, found: 336.1938.

***N*-(2-Methoxybenzyl)-2-(4-methylpiperazin-1-yl)pteridin-4-amine (18b)**. The 5-nitrosopyrimidine **17b** (0.325 g, 0.91 mmol) was heated in boiling water (2.4 mL) to give a suspension. A few drops of ethanol were added, then excess sodium dithionite until the solution became clear yellow. The cooled solution was evaporated to dryness. To the residue was added methanol (1.7 mL) then aqueous 40% glyoxal (0.125 mL, 1.1 mmol) followed by hydrochloric acid (2 M, 5 drops). The mixture was heated at reflux for 7 h, or until completion of the reaction as indicated by t.l.c. The yellow solution was extracted with a mixture of 2:1 chloroform:water. After further extraction with chloroform (3 x 30 mL), the combined organic layers were washed with water, dried over MgSO_4_, filtered and evaporated to give **18b** as yellow microprisms (0.18 g, 54%). Preparative t.l.c (94:3:3 ethyl acetate:methanol:conc. ammonia) on 30 mg afforded pure **18b**; ν_max_ (cm^-1^) 3309 (br), 2934, 2850, 2791 (w), 1597, 1570 (s); ^1^H NMR (500 MHz, CDCl_3_) δ 8.60 (1H, d, *J*=2.0 Hz), 8.12 (1H, d, *J*=2.0 Hz), 7.35-7.25 (2H, m), 6.95-6.88 (2H, m), 4.76 (2H, d, *J*=6.1 Hz), 4.05 (4H, m), 3.88 (3H, s), 2.50 (4H, m), 2.34 (3H, s); ^13^C NMR (125 MHz, CDCl_3_) δ 160.5, 160.1, 157.8, 156.2, 150.0, 137.2, 129.8, 129.1, 126.0, 123.9, 120.6, 110.5, 55.4, 55.0, 46.2, 44.0, 40.6; HRMS *m/z* M^+^ calcd. for C_19_H_23_N_7_O 365.1959, found: 365.1962.

**2-(4-Methylpiperazin-1-yl)*-N*-phenethylpteridin-4-amine (18c)**. The 5-nitrosopyrimidine **17c** (0.31 g, 0.91 mmol) was heated in boiling water (2.4 mL) to give a suspension. A few drops of ethanol were added, then excess sodium dithionite until the solution became clear yellow. The cooled solution was evaporated to dryness. To the residue was added methanol (1.7 mL) then aqueous 40% glyoxal (0.125 mL, 1.1 mmol) followed by hydrochloric acid (2 M, 5 drops). The mixture was heated at reflux for 9 h, or until completion of the reaction as indicated by t.l.c. The yellow solution was extracted with a mixture of 2:1 chloroform:water. After further extraction with chloroform (3 x 30 mL), the combined organic layers were washed with water, dried over MgSO_4_, filtered and evaporated. Column chromatography of the residue (5:95 methanol:chloroform) afforded **18c** as orange microprisms (0.04 g, 25%), mp 127-128 °C; ν_max_ (cm^-1^) 3228, 3025, 2935, 2787, 1598 (s), 1575 (s); ^1^H NMR (500 MHz, CDCl_3_) δ 8.61 (1H, d, *J*=2.0 Hz), 8.10 (1H, d, *J*=2.0 Hz), 7.35-7.30 (2H, m), 7.26-7.20 (3H, m), 6.94 (1H, br s), 4.04 (4H, br s), 3.82 (2H, dt, *J*=7.0, 6.3 Hz), 3.00 (2H, t, *J*=7.0 Hz), 2.52-2.45 (4H, m), 2.33 (3H, s); ^13^C NMR (125 MHz, CDCl_3_) δ 160.4, 160.2, 156.2, 150.2, 138.9, 137.2, 128.8, 128.75, 126.7, 123.7, 55.2, 46.2, 44.0, 42.3, 35.4; HRMS *m/z* M^+^ calcd. for C_19_H_23_N_7_ 349.2010, found: 349.2015.

**2-(4-Methylpiperazin-1-yl)-*N*-(pyridin-3-ylmethyl)pteridin-4-amine (18d)**. The 5-nitrosopyrimidine **17d** (0.30 g, 0.90 mmol) was heated in boiling water (2.4 mL) to give a suspension. A few drops of ethanol were added followed by excess sodium dithionite until the solution became clear yellow. The cooled solution was evaporated to dryness. To the residue was added methanol (1.7 mL) then aqueous 40% glyoxal (0.125 mL, 1.1 mmol) followed by hydrochloric acid (2 M, 5 drops). The mixture was heated at reflux for 7 h, or until completion of the reaction as indicated by t.l.c. The solution was extracted with a mixture of 2:1 chloroform:water. After further extraction with chloroform (3 x 30 mL), the combined organic layers were washed with water, dried over MgSO_4_, filtered and evaporated to give **18d** as yellow microprisms (0.12 g, 39%), mp 180-190 °C; ν_max_ (cm^-1^) 3246 (br), 3033 (w), 2931, 2790, 1598, 1572; ^1^H NMR (500 MHz, CDCl_3_) δ 8.64 (2H, br s), 8.52 (1H, d, *J*=4.0 Hz), 8.13 (1H, d, *J*=1.5 Hz), 7.69 (1H, d, *J*=7.5 Hz), 7.30-7.20 (2H, m), 4.79 (2H, d, *J*=5.5 Hz), 4.00 (4H, br s), 2.45 (4H, m), 2.30 (3H, s); ^13^C NMR (125 MHz, CDCl_3_) δ 160.2, 160.15, 156.2, 150.5, 149.4, 149.1, 137.4, 135.4, 133.8, 123.7, 123.4, 55.0, 46.1, 44.0, 42.4; HRMS *m/z* M^+^ calcd. for C_17_H_20_N_8_ 336.1805, found: 336.1809.

***N*-(Furan-2-ylmethyl)-2-(4-methylpiperazin-1-yl)pteridin-4-amine (18e)**. The 5-nitrosopyrimidine **17e** (0.40 g, 1.25 mmol) was heated in boiling water (3.3 mL) to give a suspension. A few drops of ethanol were added followed by excess sodium dithionite until the solution became clear yellow. The cooled solution was evaporated to dryness. To the residue was added methanol (2.4 mL) then aqueous 40% glyoxal (0.17 mL, 1.4 mmol) followed by hydrochloric acid (2 M, 5 drops). The mixture was heated at reflux for 7 h, or until completion of the reaction as indicated by t.l.c. The solution was extracted with a mixture of 2:1 chloroform:water. After further extraction with chloroform (3 x 30 mL), the combined organic layers were washed with water, dried over MgSO_4_, filtered and evaporated. Column chromatography of the residue (5:95 methanol:chloroform) afforded **18e** as yellow microprisms (0.17 g, 42%), mp 174-175 °C; ν_max_ (cm^-1^) 3237, 3134 (w), 2958, 2928, 2791, 1600 (s), 1575 (s); ^1^H NMR (500 MHz, CDCl_3_) δ 8.63 (1H, d, *J*=2.0 Hz), 8.13 (1H, d, *J*=2.0 Hz), 7.37 (1H, dd, *J*=1.9, 0.9 Hz), 7.12 (1H, t, *J*=4.8 Hz), 6.33 (1H, dd, *J*=3.3, 1.9 Hz), 6.29 (1H, d, *J*=3.3 Hz), 4.75 (2H, d, *J*=5.6 Hz), 4.03 (4H, m), 2.46 (4H, m), 2.32 (3H, s); ^13^C NMR (125 MHz, CDCl_3_) δ 160.2, 159.9, 156.2, 151.0, 150.3, 142.2, 137.3, 123.5, 110.6, 107.8, 55.0, 46.2, 44.1, 37.9; HRMS *m/z* [M+H]^+^ calcd. for C_16_H_20_N_7_O 326.1736, found: 326.1729.

**2-(4-Methylpiperazin-1-yl)-*N*-(thiophen-2-ylmethyl)pteridin-4-amine (18f)**. The 5-nitrosopyrimidine **17f** (0.406 g, 1.22 mmol) was heated in boiling water (3.2 mL) to give a suspension. A few drops of ethanol were added followed by excess sodium dithionite until the solution became clear yellow. The cooled solution was evaporated to dryness. To the residue was added methanol (2.3 mL) then aqueous 40% glyoxal (0.17 mL, 1.4 mmol) followed by hydrochloric acid (2 M, 5 drops). The mixture was heated at reflux for 7 h, or until completion of the reaction as indicated by t.l.c. The yellow solution was extracted with a mixture of 2:1 chloroform:water. After further extraction with chloroform (3 x 30 mL), the combined organic layers were washed with water, dried over MgSO_4_, filtered and evaporated. Column chromatography of the residue (5:95 methanol:chloroform) afforded **18f** as yellow microprisms (0.121 g, 29%), mp 193-194 °C; ν_max_ (cm^-1^) 3238, 3135 (w), 2928, 2847 (w), 2791, 1600 (s), 1575 (s); ^1^H NMR (500 MHz, CDCl_3_) δ 8.64 (1H, d, *J*=2.0 Hz), 8.13 (1H, d, *J*=2.0 Hz), 7.23 (1H, m), 7.19 (1H, t, *J*=6.0 Hz), 7.05 (1H, m), 4.93 (2H, d, *J*=6.0 Hz), 4.06 (4H, m), 2.48 (4H, m), 2.34 (3H, s); ^13^C NMR (125 MHz, CDCl_3_) δ 160.2, 159.8, 156.3, 150.4, 140.6, 137.3, 126.9, 126.4, 125.5, 123.5, 55.2, 46.3, 44.1, 39.6; HRMS *m/z* M^+^ calcd. for C_16_H_19_N_7_S 341.1417, found: 341.1417.

**6,7-Dimethyl-2-(4-methylpiperazin-1-yl)*-N*-(thiophen-2-ylmethyl)pteridin-4-amine (18g)**. The 5-nitrosopyrimidine **17f** (0.303 g, 0.91 mmol) was heated in boiling water (2.4 mL) to give a suspension. A few drops of ethanol were added followed by excess sodium dithionite until the solution became clear yellow. The cooled solution was evaporated to dryness. To the residue was added methanol (1.7 mL) then 2,3-butanedione (0.96 mL, 1.1 mmol) followed by hydrochloric acid (2 M, 5 drops). The mixture was heated at reflux for 7 h, or until completion of the reaction as indicated by t.l.c. The yellow solution was extracted with a mixture of 2:1 chloroform:water. After further extraction with chloroform (3 x 30 mL), the combined organic layers were washed with water, dried over MgSO_4_, filtered and evaporated to give **18g** as yellow microprisms (0.21 g, 62%), mp 89-90 °C; ν_max_ (cm^-1^) 3376, 2922, 2851 (w), 2793 (w), 1593, 1571 (s), 1558 (s); ^1^H NMR (500 MHz, CDCl_3_) δ 7.22 (1H, dd, *J*=5.0 Hz), 7.12 (1H, t, *J*=5.3 Hz), 7.05 (1H, d, *J*=3.0 Hz), 6.96 (1H, m Hz), 4.92 (2H, d, *J*=5.5 Hz), 4.16 (4H, br s), 2.69 (4H, br s), 2.51 (3H, s), 2.49 (3H, s), 2.39 (3H, s); ^13^C NMR (125 MHz, CDCl_3_) δ 159.8, 159.7, 159.6, 154.3, 146.4, 141.1, 126.8, 126.2, 125.4, 119.9, 54.8, 45.6, 43.2, 39.5, 23.3, 21.9; HRMS *m/z* M^+^ calcd. for C_18_H_23_N_7_S 369.1730, found: 369.1732.

**2-(4-Ethylpiperazin-1-yl)-5-nitroso-*N*-(thiophen-2-ylmethyl)-pyrimidine-4,6-diamine (19a)**. To nitrosopyrimidine **16f** (0.61 g, 2.1 mmol) in ethanol (2.0 mL) was added 1-ethylpiperazine (1.09 mL, 8.6 mmol). The mixture was heated at reflux for 2 h. Water (2.0 mL) was then added and the mixture again heated under reflux for a further 1 h. The solution was immediately and rapidly cooled in ice to give small reddish-violet prisms. The solution was immediately and rapidly cooled in ice to give fine dark reddish violet prisms. After standing overnight at 4 °C, filtration, washing with water and drying under vacuum afforded **19a** as fine reddish-violet prisms (0.414 g, 57%), mp 194-195 °C; ν_max_ (cm^-1^) 3248, 3195, 2934, 2796, 1598 (s); ^1^H NMR (500 MHz, CDCl_3_) rotamer A: δ 11.57 (1H, br s), 7.18 (2H, m), 6.95-6.90 (2H, m), 5.30 (1H, br s), 4.77 (2H, d, *J*=6.0 Hz), 4.10-3.90 (4H, m), 2.60-2.40 (6H, m), 1.11 (3H, t, *J*=7.0 Hz); rotamer B: δ 10.50 (1H, d, *J*=4.5 Hz), 7.85 (1H, t, *J*=6.0 Hz), 7.21 (1H, dd, *J*=5.0, 1.0 Hz), 7.02 (1H, d, *J*=3.5 Hz), 6.95-6.90 (1H, m), 5.58 (1H, br s), 4.92 (2H, d, *J*=6.0 Hz), 4.10-3.90 (4H, m), 2.60-2.40 (6H, m), 1.11 (3H, t, *J*=7.0 Hz); ^13^C NMR (125 MHz, CDCl_3_) δ 165.7, 162.4, 160.95, 160.9, 150.8, 149.9, 140.7, 140.3, 136.9, 136.6, 126.8, 126.7, 126.3, 126.25, 125.45, 125.35, 52.85, 52.8, 52.75, 52.7, 52.3, 44.5, 44.4, 44.3, 44.2, 39.0, 38.2, 12.0; HRMS *m/z* [M+H]^+^ calcd. for C_15_H_22_N_7_OS 348.1607, found: 348.1615.

**2-(4-Methyl-1,4-diazepan-1-yl)-5-nitroso*-N*-(thiophen-2-ylmethyl)pyrimidine-4,6-diamine (19b)**. To 5-nitrosopyrimidine **16f** (0.61 g, 2.1 mmol) in ethanol (2.0 mL) was added 1-methyl-1,4-diazepane (1.07 mL, 8.6 mmol). The mixture was heated at reflux for 2 h. Water (2.0 mL) was then added and the mixture again heated at reflux for a further 1 h. Evaporation of the solution gave a residue that was purified by column chromatography (1:19 methanol:chloroform) to give **19b** as dark red microprisms (0.20 g, 27%), mp 168-169 °C; ν_max_ (cm^-1^) 3250, 3197, 2934, 2795, 1600 (s); ^1^H NMR (400 MHz, DMSO-*d*_6_, 27 °C) rotamer A: δ 11.57 (1H, t, *J*=3.0 Hz), 8.21 (1H, br s), 7.50 (1H, s), 7.40-7.30 (1H, m), 7.03 (1H, d, *J*=2.0 Hz), 6.96 (1H, m), 4.78 (2H, d, *J*=5.3 Hz), 3.95-3.80 (6H, m), 2.64 (2H, m), 2.29 (3H, s), 1.84 (2H, m); rotamer B: δ 10.33 (1H, br s), 9.36 (1H, t, *J*=3.0 Hz), 7.89 (1H, s), 7.40-7.30 (1H, m), 7.06 (1H, d, *J*=2.0 Hz), 6.96 (1H, m), 4.86 (2H, d, *J*=5.6 Hz), 3.95-3.80 (6H, m), 2.64 (2H, m), 2.29 (3H, s), 1.84 (2H, m); ^13^C NMR (125 MHz, DMSO-*d*_6_) (two rotamers) δ 165.2, 165.1, 162.0, 161.9, 161.0, 160.8, 150.15, 150.1, 148.9, 148.8, 142.5, 142.4, 137.2, 136.6, 126.6, 126.4, 126.3, 126.2, 125.9, 125.7, 125.55, 125.5, 125.1, 125.0, 57.4, 57.3, 57.2, 57.1, 56.4, 56.3, 56.05, 56.0, 46.6, 46.3, 46.0, 45.95, 38.4, 37.2, 37.15, 26.9, 26.85, 26.8, 26.7; HRMS *m/z* [M+H]^+^ calcd. for C_15_H_22_N_7_OS 348.1607, found: 348.1612.

**2-(4-Ethylpiperazin-1-yl)*-N*-(thiophen-2-ylmethyl)pteridin-4-amine (20a)**. The 5-nitrosopyrimidine **19a** (0.347 g, 1.0 mmol) was heated in boiling water (2.6 mL) to give a suspension. A few drops of ethanol were added followed by excess sodium dithionite until the solution became clear yellow. The cooled solution was evaporated to dryness. To the residue was added methanol (1.9 mL) then aqueous 40% 2,3-butanedione (0.15 mL, 1.3 mmol) followed by hydrochloric acid (2 M, 5 drops). The mixture was heated at reflux for 7 h, or until completion of the reaction as indicated by t.l.c. The yellow solution was extracted with a mixture of 2:1 chloroform:water. After further extraction with chloroform (3 x 30 mL), the combined organic layers were washed with water, dried over MgSO_4_, filtered and evaporated. The residue was purified by column chromatography to give **20a** as yellow microprisms (0.11 g, 30%), mp 148-149 °C; ν_max_ (cm^-1^) 3248, 3197, 2935, 2796, 1600 (s); ^1^H NMR (500 MHz, CDCl_3_) δ 8.62 (1H, d, *J*=2.0 Hz), 8.10 (1H, d, *J*=2.0 Hz), 7.22 (2H, m), 7.04 (1H, d, *J*=3.0 Hz), 6.94 (1H, dq, *J*=5.0, 3.0 Hz), 4.92 (2H, d, *J*=5.5 Hz), 4.06 (4H, br s), 2.52 (4H, m), 2.45 (2H, q, *J*=7.0 Hz), 1.11 (3H, t, *J*=7.0 Hz); ^13^C NMR (125 MHz, CDCl_3_) δ 160.1, 159.8, 156.3, 150.3, 140.6, 137.3, 126.8, 126.4, 125.5, 123.6, 53.0, 52.5, 44.1, 39.6, 12.1; HRMS *m/z* M^+^ calcd. for C_17_H_21_N_7_S 355.1574, found: 355.1577.

**2-(4-Methyl-1,4-diazepan-1-yl)*-N*-(thiophen-2-ylmethyl)pteridin-4-amine (20b)**. The 5-nitrosopyrimidine **19b** (0.156 g, 0.45 mmol) was heated in boiling water (1.2 mL) to give a suspension. A few drops of ethanol were added followed by excess sodium dithionite until the solution became clear yellow. The cooled solution was evaporated to dryness. To the residue was added methanol (0.86 mL) then aqueous 40% 2,3-butanedione (0.067 mL, 0.58 mmol) followed by hydrochloric acid (2 M, 5 drops). The mixture was heated at reflux for 7 h, or until completion of the reaction as indicated by t.l.c. The yellow solution was extracted with a mixture of 2:1 chloroform:water (30 mL). After further extraction with chloroform (3 x 30 mL), the combined organic layers were washed with water, dried over MgSO_4_, filtered and evaporated to give **20b** as yellow microprisms (0.045 g, 28%), mp 130-131 °C; ν_max_ (cm^-1^) 3248, 3196, 2934, 2795, 1600 (s), 1537; ^1^H NMR (400 MHz, CDCl_3_, 60 °C) δ 8.62 (1H, d, *J*=2.0 Hz), 8.12 (1H, d, *J*=2.0 Hz), 7.22 (1H, dd, *J*=6.0, 1.0 Hz), 7.20 (1H, br s), 7.06 (1H, dd, *J*=5.0, 1.0 Hz), 6.96 (1H, dd, *J*=6.0, 5.0 Hz), 4.95 (2H, d, *J*=7.5 Hz), 4.12 (2H, m), 4.04 (2H, t, *J*=7.0 Hz), 2.80 (2H, br s), 2.67 (2H, m), 2.44 (3H, s), 2.01 (2H, quintet, *J*=7.0 Hz); ^13^C NMR (125 MHz, CDCl_3_, 27 °C) rotamers δ 160.5, 159.7, 159.6, 150.3, 140.9, 140.8, 137.1, 137.05, 126.9, 126.3, 126.1, 125.4, 125.3, 123.5, 58.6, 58.2, 57.1, 57.0, 46.5, 46.4, 46.2, 46.0, 39.6, 39.55, 27.2, 27.0; HRMS *m/z* [M+H]^+^ calcd. for C_17_H_22_N_7_S 356.1657, found: 356.1649.

**2-(4-Methylpiperazin-1-yl)-*N^7^*-(thiophen-2-ylmethyl)-3*H*-[1,2,3]-triazolo[4,5-*d*]pyrimidin-7-amine (21)**. A suspension of the 5-nitrosopyrimidine **17f** (0.167 g, 0.50 mmol) and 10% palladium on carbon (20 mg) in ethanol (2.5 mL) was degassed and then placed under a blanket of hydrogen. Progress of the reaction was monitored by t.l.c., and when complete the mixture was degassed, nitrogen admitted, and the process repeated three times. The mixture was then filtered through celite and evaporated. To the residue was added glacial acetic acid (2.0 mL) and then aqueous sodium nitrite (1M, 0.6 mL) was added to the stirred solution. The stirred mixture was heated at 90 °C for 2 h. Saturated aqueous sodium hydrogen carbonate was added to the cooled solution until alkaline, and the mixture then extracted ethyl acetate (4 x 30 mL). The combined organic layers were washed with water, dried over MgSO_4_, filtered and evaporated. Column chromatography of the residue (gradient from pure ethyl acetate to 80:10:10 ethyl acetate:methanol:0.880 aqueous ammonia) afforded **21** as pale orange microprisms (0.025 g, 15%), mp 185-186 °C; ν_max_ (cm^-1^) 3249, 3071, 2798 (s), 1666 (s), 1609 (s), 1580 (s); ^1^H NMR (500 MHz, CDCl_3_) δ 7.23 (1H, m), 7.16 (1H, m), 6.93 (1H, m), 5.80 (2H, br s), 5.71 (2H, s), 3.91 (4H, br s), 2.46 (4H, m), 2.33 (3H, s); ^13^C NMR (125 MHz, CDCl_3_) δ 160.9, 155.4, 151.4, 137.3, 127.8. 127.0, 126.4, 120.5, 55.0, 46.3, 44.3, 44.0; HRMS *m/z* M^+^ calcd. for C_14_H_18_N_8_S 330.1370, found: 330.1374.

**2-(4-Methylpiperazin-1-yl)-*N*-(thiophen-2-ylmethyl)-[1,2,5]thiadiazolo[3,4-*d*]pyrimidin-4-amine (22)**. To a stirred of solution of the 5-nitrosopyrimidine **17f** (0.167 g, 0.50 mmol) in aqueous 20% acetic acid (2.0 mL) maintained at 90 °C was added in portions sodium thiosulfate pentahydrate (0.31 g, 1.25 mmol). After heating the mixture at 90 °C for 1.5 h or until the reaction was complete, as monitored by t.l.c. Saturated aqueous sodium hydrogen carbonate was added to the cooled solution until alkaline, and the mixture then extracted ethyl acetate (4 x 30 mL). The combined organic layers were washed with water, dried over MgSO_4_, filtered and evaporated. To give **22** as yellow microprisms (0.12 g, 69%), mp 203-204 °C; ν_max_ (cm^-1^) 3309 (br), 3105 (w), 2930, 2791, 1618, 1600, 1530 (s); ^1^H NMR (500 MHz, DMSO-*d*_6_) δ 9.52 (1H, t, *J*=6.0 Hz), 7.38 (1H, m), 7.09 (1H, m), 6.96 (1H, m), 4.83 (2H, d, *J*=6.0 Hz), 4.02 (4H, m), 2.94 (4H, m), 2.49 (3H, s); ^13^C NMR (125 MHz, DMSO-*d*_6_) δ 166.0, 161.2, 153.9, 141.0, 135.1, 126.5 (2 lines), 125.5, 53.1, 43.6, 42.3, 38.7; HRMS *m/z* [M+H]^+^ calcd. for C_14_H_18_N_7_S_2_ 348.1065, found: 348.1055.

**2-(4-Methylpiperazin-1-yl)-*N*-(thiophen-2-ylmethyl)-[1,2,5]oxadiazolo[3,4-*d*]pyrimidin-4-amine (23)**. To a stirred mixture of 5-nitrosopyrimidine **17f** (0.167 g, 0.50 mmol) in acetic acid (2.0 mL) under nitrogen was added lead tetraacetate (0.266 g, 0.60 mmol). The resulting suspension was stirred under nitrogen for 4 h. Acetic acid was removed from the yellow solution was evaporated to dryness. The residue was stirred with saturated aqueous sodium hydrogen carbonate and the alkaline suspension filtered and washed with water (5 x 20 mL). Column chromatography of the dried residue (5:95 methanol:chloroform) afforded **23** as yellow microprisms (0.016 g, 10%), mp 178-179 °C; ν_max_ (cm^-1^) 3246 (br), 2931, 2797, 1665, 1575 (s); ^1^H NMR (500 MHz, CD_3_OD) δ 7.23 (1H, d, *J*=5.5 Hz), 7.10 (1H, d, *J*=3.0 Hz), 6.93 (1H, dd, *J*=5.5, 3.0 Hz), 4.90 (2H, m), 4.00 (4H, m), 2.46 (4H, m), 2.32 (3H, s); ^13^C NMR (125 MHz, CD_3_OD) δ 163.4, 162.7, 153.4, 141.3, 136.2, 127.7, 127.6, 126.4, 55.8, 46.0, 45.9, 40.2; HRMS *m/z* M^+^ calcd. for C_14_H_17_N_7_OS 331.1210, found: 331.1216.

**2. Experiments *in silico***

**Molecular docking simulations for** **soybean lipoxygenase**

Visualization of the protein (PDB code: 3PZW) was performed using UCSF Chimera [42]. Water molecules were removed, missing residues were added with Modeller [43], hydrogen atoms and AMBER99SB-ILDN charges were added, and the charge on iron was set to +2.0, with no restraint applied to the iron atom and the ligands. Ligand 3D coordinates were generated and minimised with OpenBabel [49] using the MMFF94 force field [50]. ACPYPE (AnteChamber PYthon Parser interfacE) [51] was used to generate ligand topologies and parameters using Antechamber [52]. Energy minimizations were carried out using the AMBER99SB-ILDN force field [53] with GROMACS 4.6.5 [54] as the molecular dynamics simulation toolkit. Docking was performed with AutoDock Vina (1.1.2) [55] using a grid box of size 18.15 Å in X, Y, Z dimensions, centred in the catalytic site, using AutoDockTools; automated docking with selective receptor flexibility, as implemented in MGLTools 1.5.6, was used to generate docking input files. PyMOL and UCSF-Chimera [44] were used to analyze docking results. Docking was carried out with an exhaustiveness value of 64 and a maximum output of 100 docking modes.

For comparison, the known, but weaker, LOX inhibitor NDGA was also docked into soybean LOX. Fig. 8 shows the superposition of the modes of binding of NDGA and **18d** to soybean LOX.


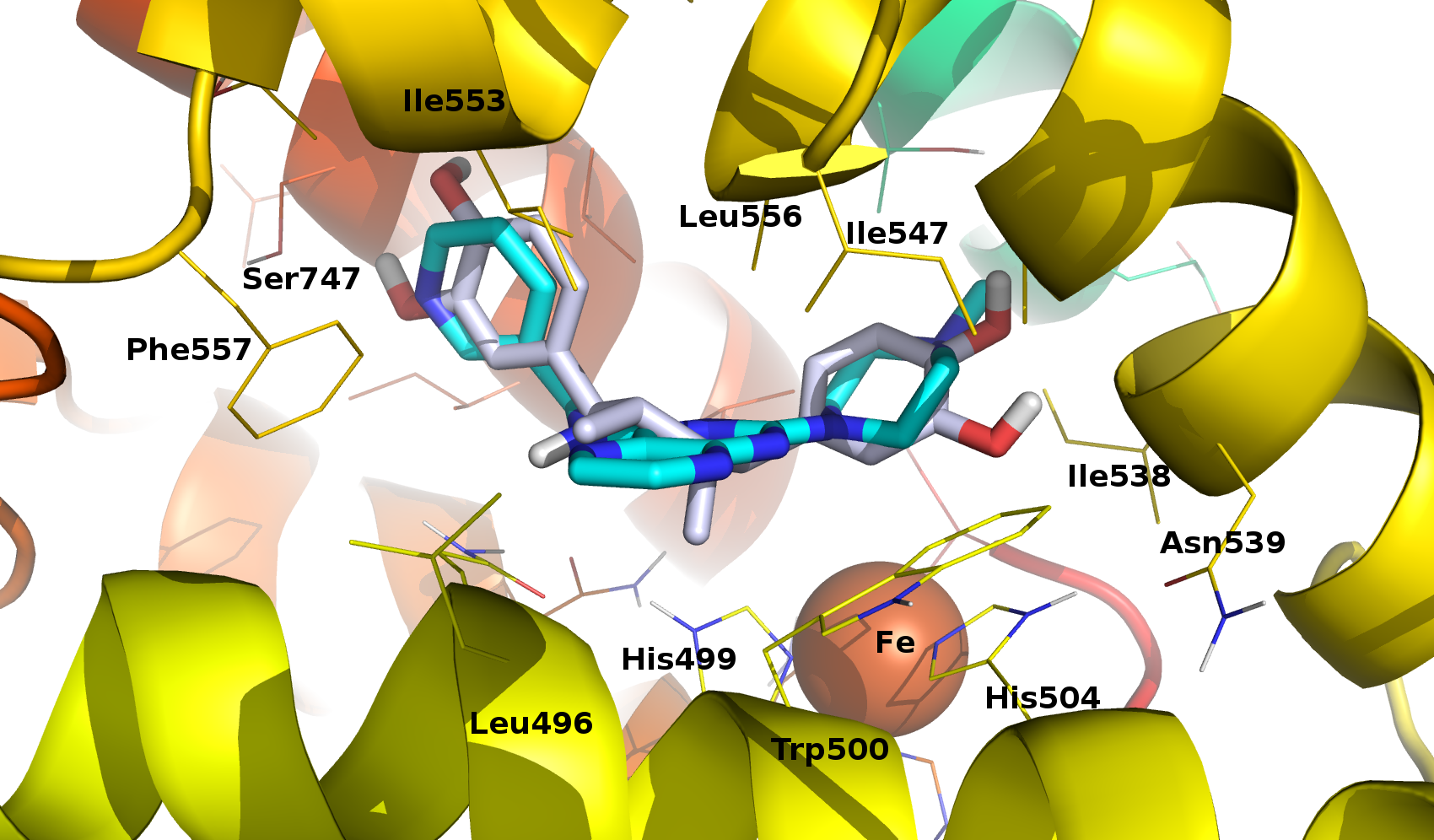


**Figure S1**

Docking pose of pteridine **18d** (depicted in cyan) and NDGA (depicted in grey) bound to soybean lipoxygenase (LOX-1) derived by modification of PDB code: 3PZW. Energy minimizations were carried out using the AMBER99SB-ILDN force field [42] with GROMACS as the molecular simulation toolkit [43]. AutoDock Vina (1.1.2) [42] was used for docking. Iron is rendered as a red sphere. Figure 8, prepared using PyMOL, represents the preferred pose according to scoring function.

**3. Experiments *in vitro***

For *in vitro* assays each experiment was performed at least in triplicate. The standard deviation of absorbance was less than 10% of the mean.

**Determination of soybean lipoxygenase inhibition** [34, 35]

A solution in DMSO of each compound to be tested was incubated at room temperature with sodium linoleate (0.1 mM) and a solution of soybean lipoxygenase (EC 1.13.11.12) (1:9 x 10^4^ w/v in saline, 0.2 mL, Sigma). The conversion of sodium linoleate to 13-hydroperoxylinoleic acid was monitored by observation at 234 nm and compared with the value of appropriate standard inhibitor (Table 1). Concentrations from 0.01 μM -100 μM were used for the determination of IC_50_ values.

**Determination of reducing activity using 1,1-diphenyl-2-picrylhydrazyl radical (DPPH)** [34, 35]

To a solution of DPPH in absolute ethanol was added an equal volume of the compound to be tested dissolved in DMSO (20 µL). From a stock solution (10 mM) was prepared a final solution of 100 μM for evaluation of the compounds. The absorbance at 517 nm was recorded after 20 and 60 min at room temperature (Table 2).

**Inhibition of linoleic acid peroxidation** [34]

Production of conjugated diene hydroperoxide by oxidation of linoleic acid in an aqueous dispersion was monitored at 234 nm. 2,2’-Azobis(2-amidinopropane) dihydrochloride (AAPH) was used as a free radical initiator. This assay was used to follow oxidative changes and to determine the inhibition of the peroxidation of sodium linoleate by each tested compound. A dispersion of sodium linoleate (16 mM, 10 μL) was added to the UV cuvette containing phosphate buffer (0.05 M, 0.93 mL) at pH 7.4 and kept in a thermostat at 37 °C. The oxidation reaction was initiated at 37 °C under air by the addition of AAPH solution (40 mM, 50 μL). Oxidation was carried out in the presence of aliquots (10 μL) of the tested compound. In the assay without antioxidant, lipid oxidation was measured in the presence of DMSO (10 μL). The rate of oxidation at 37 °C was monitored by recording the increase in absorption at 234 nm caused by conjugated diene hydroperoxide formation (Table 2). Concentrations from 1 μM - 100 μM were used for the determination of IC_50_ values.

**Competition of pteridine derivatives with DMSO for hydroxyl radicals** [34, 35]

Hydroxyl radicals generated by the Fe^3+^/ascorbic acid system were detected by determination of formaldehyde produced from the oxidation of DMSO, according to the procedure of Nash [56]. The reaction mixture comprised ethylenediaminetetracetic acid (EDTA) (0.1 mM), Fe^3+^ (167 μM), DMSO (33 mM) in phosphate buffer (50 mM, pH 7.4), ascorbic acid (10 mM) and the compound to be tested at 100 μM. After incubation at 37 °C for 30 min the mixture was quenched with aqueous trichloroacetic acid (17% w/v) (Table 2) and the percentage competition of the tested compounds with DMSO for hydroxyl radicals was determined (Table 2).

**4. Experiments *in vivo***

**Materials and Methods**

Fisher 344 rats (150-200 g) were housed in wire-mesh cages, given standard laboratory chow and tap water *ad libitum* and acclimatized for one week before experiments. Rats were randomly assigned to experimental groups. All *in vivo* experiments conform to the law for the protection of experimental animals (Republic of Greece) and are registered at the Veterinary Administration of the Republic of Greece in accordance with recognized guidelines on animal experimentation including Directive 2010/63/EU.

**Induction of colitis** [57]

Rats were anesthetized using pentobarbital (50 mg/kg ip) prior to administration with 1 mL of either vehicle (0.9% w/v saline) or the compound under study at doses 0.01mmol/kg intrarectally via a polyethylene cannula (fitted to a 1 mL syringe) with the tip inserted approximately 8 cm into the anus. Following 30 min pretreatment, 1 ml of 4% acetic acid was introduced into the colon intrarectally and, after exposure for 60 s, the excess fluid was withdrawn. The luminal content was then expelled by instillation of air (10 mL), the ligature was removed and the abdomen was sutured. Both groups of rats were allowed to recover with food and water and the resulting injury was assessed after 48 h. The experiment was conducted using two groups, each containing 3 animals.

*Assessment of colonic damage.* Rats were weighed, inspected for the presence of diarrhea and sacrificed using diethyl ether. The colons were excised and opened longitudinally, rinsed with cold saline, pinned out and observed under a dissecting microscope. Colonic damage was scored using the system of MacPherson and Pfeiffer [60]: 0 – normal appearance, 1- hyperaemia, 2 – patchy petechial bleeding, 3 – diffuse petechial bleeding, 4 – partial exfoliated mucosa or single erosion or ulceration, 5 – diffuse exfoliated mucosa or multiple erosions or ulceration. The presence or absence of adhesions between the colon and the surrounding tissues was recorded. Scoring of the colonic damage (Table 4) was carried out by an observer unaware of the treatment.

**Inhibition of carrageenin-induced edema** [34]

Edema was induced in the right hind paw of Fisher 344 rats (150-200 g) by intradermal injection of aqueous carrageenin (0.1 mL, 2%). Pregnant females were excluded from the study, but both sexes were used. Each group comprised 6-15 rats which were bred and housed under standard conditions and received a diet of commercial food pellets and water *ad libitum* during maintenance but were entirely fasted during the experiment period. Each compound (0.01 mmol/kg body weight) was suspended in water to which had been added a few drops of Tween 80 and then the mixture was ground in a mortar prior to being administered intraperitoneally and simultaneously with the carrageenin injection. Rats were euthanized 1 h after injection, and the difference between the weight of the injected and non-injected paws was calculated for each animal. The change in paw weight was compared with that in control animals (treated with water) and the inhibition of the edema was expressed as a percentage (Table 4). Carrageenin paw edema data are the mean values from two independent experiments, and with a standard error of the mean less than 10 %.

**IC_50_ determinations**. OriginPro 8 was used to determine IC_50_ values from the sigmoidal line fitted to a graph of log[concentration] against the average percentage inhibition from two independent experiments, with at least 6 different concentrations. The SEM is the calculated standard error in the IC_50_ value of the fitted line.
